# Bioinspired Ultrasensitive Flexible Strain Sensors for Real-Time Wireless Detection of Liquid Leakage

**DOI:** 10.1007/s40820-024-01575-2

**Published:** 2024-11-22

**Authors:** Weilong Zhou, Yu Du, Yingying Chen, Congyuan Zhang, Xiaowei Ning, Heng Xie, Ting Wu, Jinlian Hu, Jinping Qu

**Affiliations:** 1 Key Laboratory of Material Chemistry for Energy Conversion and Storage, Ministry of Education, Hubei Key Laboratory of Material Chemistry and Service Failure and Hubei Engineering Research Center for Biomaterials and Medical Protective Materials, School of Chemistry and Chemical Engineering, Huazhong University of Science and Technology, Wuhan, 430074 People’s Republic of China; 2 School of Materials Science and Engineering, Wuhan Institute of Technology, Wuhan, 430205 People’s Republic of China; 3 Department of Biomedical Engineering, City University of Hong Kong, Hong Kong, SAR 999077 People’s Republic of China

**Keywords:** Thermoplastic polyurethane, Bioinspired, Cracks, Liquid leakage, Flexible strain sensor

## Abstract

Liquid leakage of pipeline networks not only results in considerable resource wastage but also leads to environmental pollution and ecological imbalance. In response to this global issue, a bioinspired superhydrophobic thermoplastic polyurethane/carbon nanotubes/graphene nanosheets flexible strain sensor (TCGS) has been developed using a combination of micro-extrusion compression molding and surface modification for real-time wireless detection of liquid leakage. The TCGS utilizes the synergistic effects of Archimedean spiral crack arrays and micropores, which are inspired by the remarkable sensory capabilities of scorpions. This design achieves a sensitivity of 218.13 at a strain of 2%, which is an increase of 4300%. Additionally, it demonstrates exceptional durability by withstanding over 5000 usage cycles. The robust superhydrophobicity of the TCGS significantly enhances sensitivity and stability in detecting small-scale liquid leakage, enabling precise monitoring of liquid leakage across a wide range of sizes, velocities, and compositions while issuing prompt alerts. This provides critical early warnings for both industrial pipelines and potential liquid leakage scenarios in everyday life. The development and utilization of bioinspired ultrasensitive flexible strain sensors offer an innovative and effective solution for the early wireless detection of liquid leakage.

**Supplementary Information:**

The online version contains supplementary material available at 10.1007/s40820-024-01575-2.

## Highlights

- Micro-extrusion compression molding and surface modification were proposed for the mass fabrication of a superhydrophobic thermoplastic polyurethane sensor (TCGS).
- Inspired by scorpions, TCGS features Archimedean spiral crack arrays and micropores, achieving 218.13 sensitivity at 2% strain, a 4300% increase, and over 5000 usage cycles of durability.
- The robust superhydrophobicity of TCGS improves sensitivity and stability for detecting small-scale liquid leakage, allowing precise monitoring across various sizes and compositions while providing early warnings in various scenarios.

**Supplementary Information:**

The online version contains supplementary material available at 10.1007/s40820-024-01575-2.

## Graphical abstract

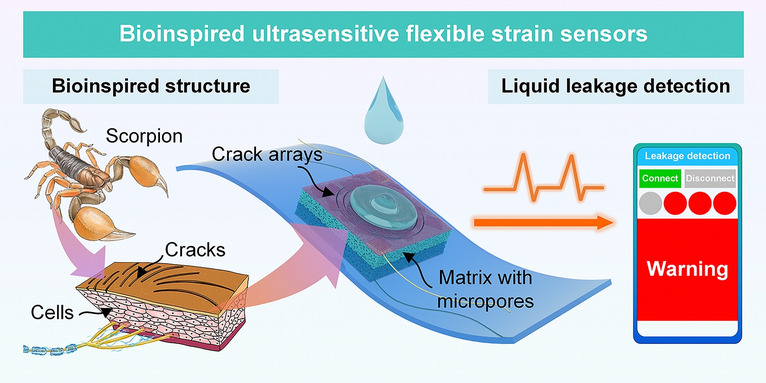

## Introduction

In contemporary urban settings, pipeline networks play a vital role in the distribution of essential resources such as potable water and industrial fluids, underpinning both the smooth operation of cities and the well-being of their inhabitants [1, 2]. Despite their significance, these infrastructures inevitably face the issue of leakages, which are a consequence of prolonged use, corrosion, natural catastrophes, or intentional tampering [3]. Such incidents not only squander valuable water resources but also pose immediate risks to public health and safety, inflict lasting damage on urban ecosystems, and could precipitate severe accidents leading to substantial economic and human losses [4, 5]. Current methods for leakage detection, which rely on specialized hardware such as acoustic devices [6, 7], fiber optic sensors [8], infrared sensors [9], and radar [10], often encounter obstacles. These include high costs, limited monitoring range, insensitivity to minor leakages, and an inability to continuously monitor in real-time. In light of these challenges, it becomes imperative to develop a novel leakage detection technology that marries high efficiency and affordability with extensive coverage and real-time remote monitoring capabilities. Such advancement is essential for bolstering urban resilience to environmental emergencies and safeguarding public safety.

In recent years, superhydrophobic flexible strain sensors have emerged as highly promising for applications in liquid leakage detection due to their remarkable flexibility and high sensitivity [11–14]. Ingeniously designed into irregular shapes, these sensors excel at detecting difficult-to-access fault locations within three-dimensional spaces [15–17]. They effectively translate the variations of external forces into the change of the electrical response signal in real-time, demonstrating extraordinary sensitivity [18–21]. Primarily constructed from polymers and conductive materials, the superhydrophobic flexible strain sensors are produced through intricate methods such as photolithography, etching, printing, and cutting, yielding a broad spectrum of sizes and shapes [22–26]. Current research endeavors are directed towards enhancing the sensors’ sensitivity, durability, and response time, and integration with wireless technology [27–31]. The enhancement of superhydrophobicity is also crucial for preserving sensor functionality in liquid environments, significantly improving the detection of minute liquid leakages [32–35]. To achieve these improvements, researchers have designed a range of micro-nanostructures, including micropores, cracks, waves, grids, and aligned patterns [36–40], and have employed advanced materials such as MXenes, graphene nanosheets (GNS), and carbon nanotubes (CNTs) to optimize performance [41–45]. However, in practical applications, finding the optimal balance among these performance indicators and scaling up the continuous production of superhydrophobic flexible strain sensors remains a significant challenge [46–48].

In response to this challenge, the remarkable sensing capabilities of organisms in nature provide invaluable inspiration [49–53]. Specifically, scorpions, despite their diminishing vision, depend on their highly developed and sensitive sensing mechanism to adeptly navigate harsh environmental conditions [54]. This mechanism is crucial to their survival, offering them essential, long-term information for living due to its high sensitivity and stability [55]. Against this backdrop, we have developed a micropore-crack synergistic superhydrophobic thermoplastic polyurethane (TPU)/CNTs/GNS sensor (TCGS) inspired by the sensory capabilities of scorpions. This sensor employs an innovative manufacturing method that combines micro-extrusion compression molding (μ-ECM) with surface modification, featuring cost-effectiveness, ease of operation, and suitability for large-scale production [56, 57]. The sensor replicates the advanced sensory mechanisms of scorpions, allowing the micropores and cracks to deform under forces applied from various directions. Such deformation, coupled with alterations in the conductive networks of surface GNS and internal CNTs, induces changes in resistance, thus providing a highly sensitive electrical response to minor stresses. Additionally, the superhydrophobic conductive surface design of TCGS significantly improves the efficiency and stability of liquid leakage detection in wet environments, ensuring exceptional sensitivity and accuracy across various liquid environments. By integrating the TCGS into a leakage detection device, this system enables real-time wireless monitoring of liquid leakage of various sizes, velocities, and compositions, covering leakage ranging from droplet to large flow, and issuing corresponding real-time alerts. This liquid leakage detection device can be broadly applied in pipeline networks, where its high sensitivity and extensive applicability not only allow for rapid response to leakage incidents and prevent resource loss but also contribute significantly to reducing safety hazards and promoting sustainable development.

## Experimental Section

### Materials

TPU (Elastollan 1185A, density $\rho$ = 1.12 g cm^−3^) was purchased from BASF Co., Ltd. Na_2_SO_4_ (#200) was provided by Sichuan Tongqing Nanfeng Co., Ltd. The microscopic morphology and particle size of Na_2_SO_4_ are shown in Fig. S1. CNTs (grade NC7000, carbon purity ≥ 98%) with an average diameter of ~ 9.5 nm and an average length of ~ 1.5 μm were purchased from Nanocyl Corporation (Belgium). GNS (grade SE1233, carbon purity ≥ 98%) with an average diameter of < 10 μm was purchased from Sixth Element (Changzhou) Materials Technology Co., Ltd. The microstructures of CNTs and GNS are shown in Fig. S2. Hexamethyl disilylamine (HMDS) was provided by Shanghai Aladdin Biochemical Technology Co., Ltd. Ethyl acetate (EA) was provided by Sinopharm chemical reagent Co., Ltd. Polydimethylsiloxane (PDMS; Sylgard 184) was obtained from Dow Corning. Archimedean spiral molds were designed by us and then fabricated by Dongguan Taixin Metal Materials Co., Ltd.

### Preparation of TC Foams

The TC foams were prepared by μ-ECM. First, the weighed TPU, Na_2_SO_4_, and CNTs were mixed in a high-speed mixer for 3 min, and the content of each component is shown in Table S1. The mixture was blended in an eccentric rotor extruder (ERE) with a rotor diameter of 33.5 mm and an eccentricity of 3 mm to prepare TPU/Na_2_SO_4_/CNTs melts (Fig. S3). The rotor speed was 35 rpm, and the temperature profile from the hopper to the die was 120, 150, 180, and 200 °C. Subsequently, the TPU/Na_2_SO_4_/CNTs melts were extruded into a mold cavity equipped with copper mesh and wires, and then the melts were squeezed into the Archimedean spiral template by the downward compression of the moving mold half. After cooling and demolding, the TPU/Na_2_SO_4_/CNTs composites featuring Archimedean spiral cracks on the surface and implanted electrodes (copper mesh and wires) were obtained. The composites were next sonicated in water for 10 h to remove the Na_2_SO_4_ component and were dried at 70 °C for 12 h to obtain the TC foams. The TC foams with cracks and implanted electrodes were named TC-$w$, where $w$ represents the $w$% concentration of CNTs in the TPU matrix. In addition, the TC foams without cracks and implanted electrodes were prepared for comparison.

### Preparation of TCGS

The TCGS was further prepared through surface modification and assembly. Initially, 0.1 g GNS, 1 g PDMS, and 1 mL HMDS were dispersed in 10 mL EA solution, and sonicated for 10 min using a split-type ultrasonic crusher (XM-1000 T, Xiaomei Ultrasonic Instrument (Kunshan) Co., Ltd.). The dispersion was then sprayed onto the TC-1 surface using an air spray gun with a spray distance of 15 cm and an air pressure of 0.6 MPa. After curing at 70 °C for 5 h and at 150 °C for 2 h, a superhydrophobic and conductive GNS/PDMS network was formed on the foam surface. Finally, the crack-free side of the foam was covered with a 1 mm thick TPU film, and the TCGS was assembled using 200 °C hot air from an electric hot air gun. TCGS was named TCGS-$N$, where $N$ represents the number of Archimedean spiral crack turns in each sensor. Additionally, TCGS-CM (without cracks and micropores) and TCGS-M (with cracks but without micropores) were also prepared for comparison.

### Characterizations

The chemical compositions of the samples were analyzed by Fourier transform infrared spectroscopy (FTIR, INVENIO-R) and X-ray photoelectron spectroscopy (XPS, Shimadzu/Krayos AXIS Ultra DLD). The crystal structures of the materials were examined using X-ray diffraction (XRD, SmartLab-SE), employing Cu-Kα radiation (40 kV, 30 mA) at room temperature and scanning at a speed of 10° min^−1^. The tensile properties of the TC foams were tested by an electric universal testing machine (UTM 4024X, Shenzhen Suns Technology Stock Co., Ltd.) at a crosshead speed of 10 mm min^−1^. The volume conductivities exceeding 10^−5^ S cm^−1^ were measured using a double electrical four-probe tester (RTS-9, 4Probes Tech Ltd.), while the volume conductivities below 10^−5^ S cm^−1^ were measured using an ultra-high resistance microcurrent tester (ST2643, Suzhou Jingge Electronic Co., Ltd.). Morphologies and elemental distributions were examined using scanning electronic microscopy (SEM, TESCAN MIRA LMS). The dispersion of the CNTs in the TPU matrix was assessed by a transmission electron microscopy (TEM, Tecnai G2 20), and ultrathin sections of 70 nm were prepared by a Leica EM UC7 ultramicrotome at − 100 °C. The surface structure of the TCGS-7 was characterized utilizing a laser scanning confocal microscopy (OLS5100). The electrical signals were recorded by a commercially portable test system (TruEbox, LinkZill company). The CAs and RAs were measured using a CA measuring instrument (JC2000D5A, Shanghai Zhongchen Digital Technology Apparatus Co., Ltd.). Dynamics of water droplet impact were captured by a high-speed camera (UX100, Photron. Ltd.) at a frame rate of 10,000 fps.

## Results and Discussions

### Design and Fabrication of TCGS

**Fig. 1:**
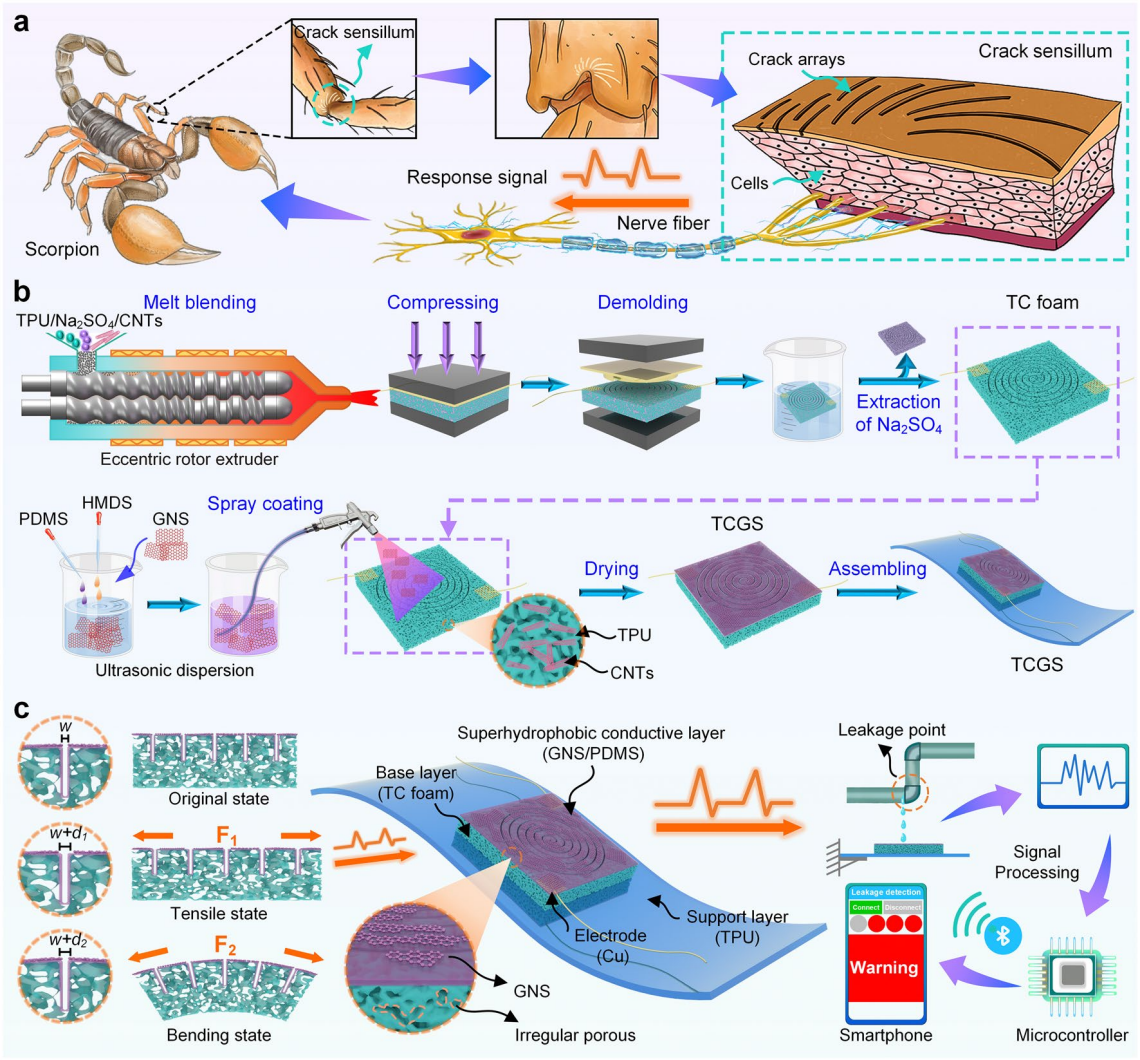
Schematic illustrations of TCGS inspired by the scorpion sensory system. **a** Scorpions possess ultrasensitive crack organs that help them perceive external forces and vibrations. The enlarged image depicts the sensory system, which consists of crack arrays, numerous cells, and neurons. **b** Illustration showing the preparation procedure of TCGS. **c** Application of TCGS in real-time wireless detection of liquid leakage

Scorpions are sensitive to vibrations around them via the curved crack sensillum near their leg joints between the tarsus and basitarsus [58]. As shown in Figs. 1a and S4, the crack sensillum comprises approximately twelve curved cracks on its surface, accompanied by numerous cells beneath. The arrangement of crack arrays and cell groups enables ultrasensitive displacement detection by allowing for omnidirectional mechanical compliance, which leads to the deformation of the crack sensillum in response to minor external force variations from any direction. The signals produced by the deformation are transmitted and processed through the neuronal system, enabling the scorpion to acquire survival information, such as the presence of water, prey, or threats. Inspired by this ability of scorpions, we designed a TCGS by combining industrialized μ-ECM and surface modification. Figure 1b illustrates the two main steps included in the preparation process: (i) the preparation of the TPU/CNTs (TC) foam by μ-ECM, and (ii) the preparation of the TCGS by surface modification and assembly. The TCGS consisted of three layers: a superhydrophobic conductive layer made of GNS and PDMS, a micropore-crack synergistic conductive TC foam with implanted electrodes, and a TPU film support layer (Fig. 1c). When the TCGS is subjected to stretching or bending, its micropores and cracks deform accordingly. These deformations cause changes in the conductive networks of the GNS on the sensor’s surface and the CNTs internally, leading to variations in resistance. The microcontroller captures these resistance changes through electrodes embedded in the sensor and analyzes them to accurately identify the behavior of external forces. By transmitting the signals to a smartphone via Bluetooth or WiFi, a stable and highly sensitive remote monitoring device for liquid leakage can be established.

### Structure and Characterization of TCGS

**Fig. 2:**
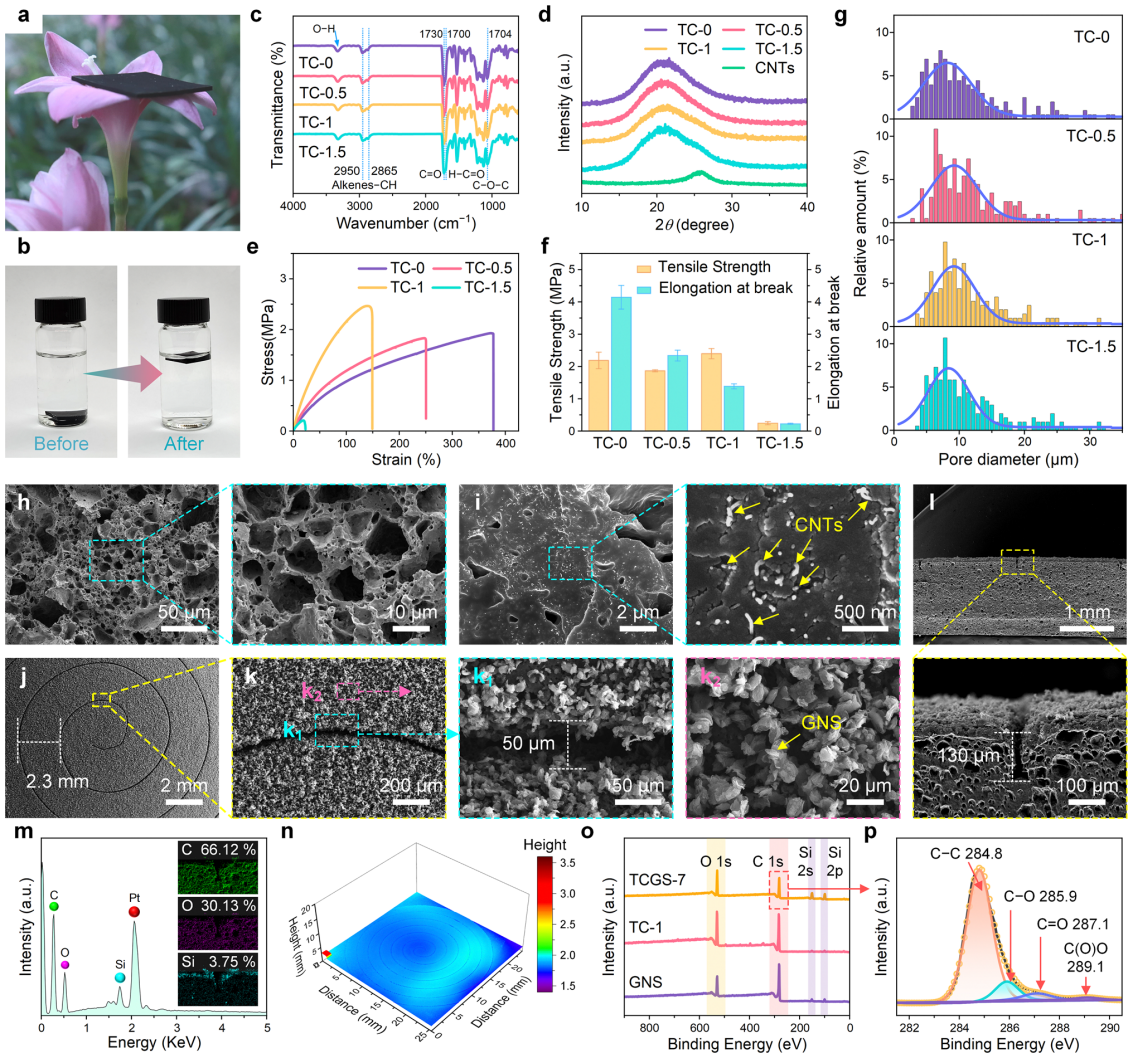
Microscopic morphology and characterization of TCGS. **a** A square TC-1 supported by a pink flower. **b** TC-1 shown before and after immersion in deionized water for 12 h. **c** FTIR spectra. **d** XRD patterns of TC-0, TC-0.5, TC-1, TC-1.5, and CNTs. **e** Tensile stress–strain curves. **f** Tensile strength and elongation at break for TC foams. **g** Pore size distribution of TC foams. **h** Cross-sectional SEM images of TC-1. **i** Enlarged view of **h, j, k** Surface SEM images of TCGS-7. **l** Cross-section SEM images and **m** elemental distribution of TCGS-7. **n** Laser scanning confocal microscopy image. **o** XPS survey spectra of GNS, TC-1, and TCGS-7. **p** C 1*s* XPS spectrum of TCGS-7

The TCGS is structured at three levels: the micropores, the Archimedean spiral crack arrays, and the GNS/PDMS coating on the surface. The TC-1, with dimensions of 40 × 40 × 1 mm^3^, is lightweight with a density of 0.586 g cm^−3^, and can easily be supported by a pink flower (Figs. 2a and S5). Photographs of the TC-1 taken before and after 12 h of immersion in deionized water reveal that the water remained clear after the Na_2_SO_4_ component had been completely removed (Figs. 2b and S6), indicating that all CNTs are preferentially distributed within the TPU skeletons. The actual weight loss ratios of TC foams are consistent with theoretical predictions (Fig. S7), confirming that the Na_2_SO_4_ component has been completely removed, forming a continuous porous skeleton. The interaction between TPU and CNTs was investigated using FTIR spectroscopy, as shown in Fig. 2c. As the concentration of CNTs increases, the hydroxyl peak of TC-0 to TC-1.5 shifts from 3328.9 to 3321.2 cm^−1^, which is attributed to the hydrogen bonding between TPU and CNTs [59]. Figure 2d shows the XRD patterns of TC-0, TC-0.5, TC-1, TC-1.5, and CNTs. The diffraction peak for TC-0, which ranges from 14° to 29° and is centered at 2 $\theta$ = 20.9°, indicates the presence of both crystalline and amorphous phases in TPU. The addition of different concentrations of CNTs does not affect the crystallization of TPU, as evidenced by the consistent XRD patterns across all samples [60]. As the concentration of CNTs increases, the tensile strength of the TC foams initially decreases then increases, and finally decreases again (Fig. 2e, f). The TC-1 exhibits the best mechanical strength (2.40 MPa) and high elongation at break (138.52%), indicating the most significant reinforcing effect of CNTs within the TPU matrix. However, in the TC-1.5, these properties drop sharply to 0.24 MPa and 22.38%, respectively. This decline is attributed to the aggregation of CNTs at high concentrations, leading to increased structural defects and weakened interfacial bonding, thereby reducing mechanical performance. Additionally, with increasing CNTs concentration, more conductive networks form within the TPU matrix, leading to a steady increase in electrical conductivity, with the TC-1 achieving 2.33 × 10^−5^ S cm^−1^ (Fig. S8). As shown in Figs. 2g, h and S9, the TC foams possess interconnected irregular micropores. In the TC-1, these micropores range from 3.12 to 51.7 μm, with an average size of 12.29 μm. These micropores reduce the weight of the sensors, enhancing usability, and allowing deformation under multidirectional forces. These results indicate that the volumetric extensional flow in the ERE achieves a homogeneous dispersion of Na_2_SO_4_ and CNTs within the TPU matrix, forming a stable three-dimensional interconnected conductive network with uniform pore size distribution (Fig. S3 and Note S1) [56]. After evaluating the combined properties of the TC foams, the TC-1 was selected as the primary body for the TCGS due to its excellent mechanical strength, conductivity, and uniform pore size distribution.

In the TCGS, the uniformly dispersed CNTs build interconnected conductive channels within the TPU skeleton (Figs. 2i and S10). These channels can accommodate deformations in all directions and have a broad strain range, allowing them to sense external forces coming from all directions. The metal templates featuring Archimedean spirals with varying numbers of turns were designed to construct secondary crack arrays (Fig. S11). In the TCGS-7, the crack arrays are arranged in a 7-turn Archimedean spiral (Fig. 2j, k), with a crack spacing of approximately 2.3 mm and a crack width of 50 µm. Additionally, Figs. S12 and S13 show SEM images of TCGS-7, featuring cracks with widths of 25, 100, and 127 µm, as well as depths of 65 and 155 μm, respectively. The GNS/PDMS coating is uniformly distributed on the TCGS-7 surface, forming a tertiary micro-nanostructured layer with superior electrical conductivity and superhydrophobicity, which can effectively shield the TCGS-7 from damage caused by water droplets or flow (Fig. S8). The Archimedean spiral-shaped cracks can produce displacement responses to external forces in all directions, causing changes in the conductive network formed by GNS on the surface and variations in resistance. Moreover, the cracks, with a depth of around 130 µm, are surrounded by numerous micropores (Fig. 2l). These micropores can disperse the stress concentration induced by the cracks and improve the stability. The cross-sectional elemental distribution of TCGS-7 was further analyzed, and the results are shown in Fig. 2m. Dense carbon and oxygen are distributed in the matrix, while silicon is predominantly located on the surface, reflecting the uniform distribution of GNS bonded to PDMS. This indicates that the GNS/PDMS layer primarily forms on the surface and does not penetrate the cracks, thereby effectively preserving structural integrity. The image taken with laser scanning confocal microscopy reveals that the TCGS-7 surface is geometrically homogeneous. The cracks are uniformly distributed in an Archimedean spiral, ensuring the stability of the sensing signal (Fig. 2n). The surface composition of GNS, TC-1, and TCGS-7 was analyzed using XPS spectroscopy (Fig. 2o). Significant increases in the intensities of the O 1*s*, Si 2*s*, and Si 2*p* peaks indicate the successful construction of the GNS/PDMS coating on the surface of TCGS-7. Figure 2p shows the high-resolution XPS spectra of carbon in the TCGS-7. The characteristic peaks at 284.8, 285.9, 287.1, and 289.1 eV belong to C − C, C − O, C = O, and C(O)O, respectively. Compared to TC-1, the decrease in the carbon–oxygen ratio and the intensity of the C-O peak in TCGS-7 indicates that GNS is partially embedded in PDMS, forming a dense and uniform GNS/PDMS coating. This coating increases the surface roughness of TCGS-7 and reduces its surface free energy, contributing to the stable superhydrophobicity of TCGS-7 (Table S2 and Fig. S14). Therefore, the synergistic structure of micro-pores and cracks, along with the stable internal and external three-dimensional conductive network, provides a highly sensitive and reliable material foundation for liquid leakage detection.

### Strain Sensing Performance

**Fig. 3:**
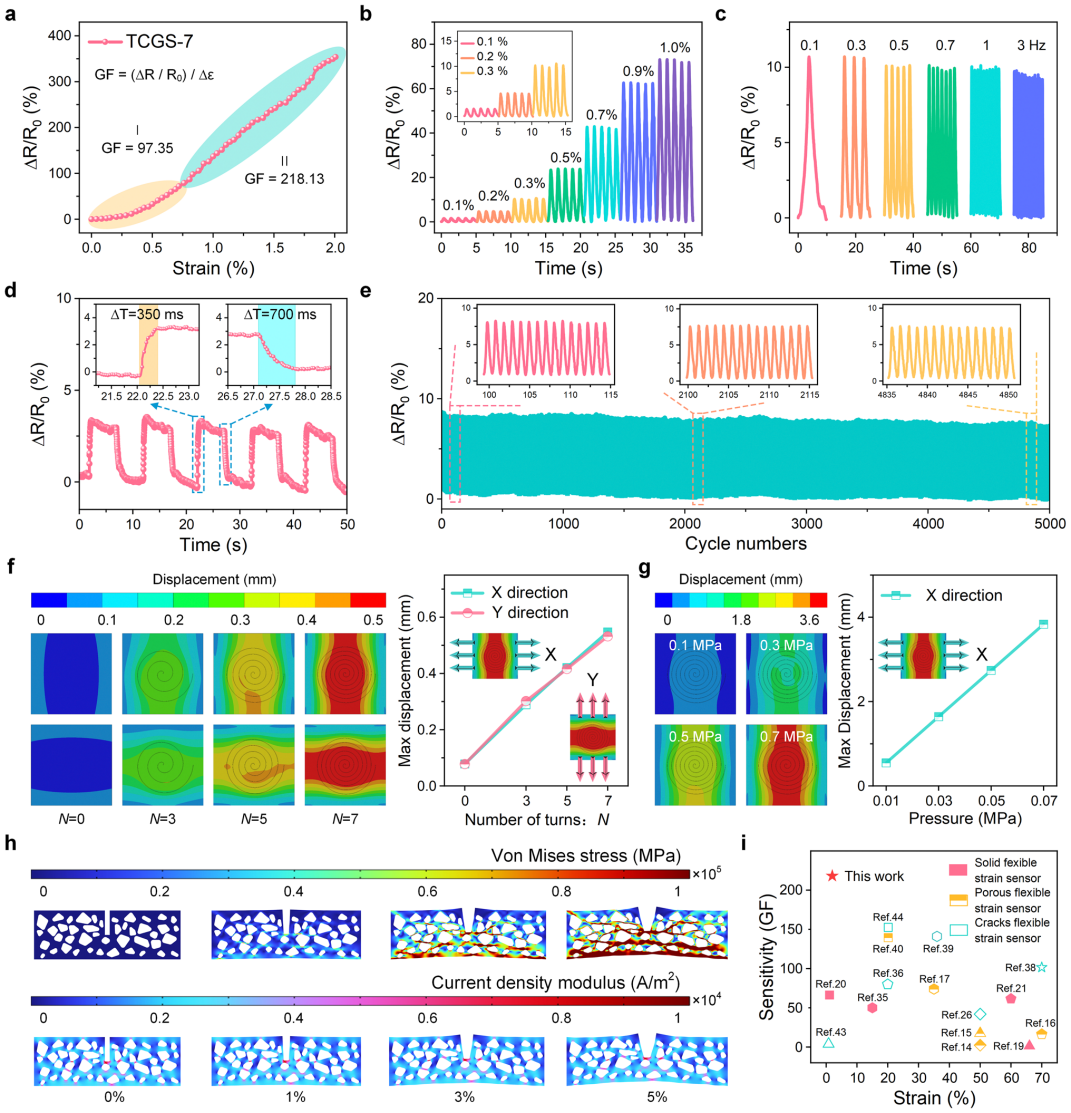
Working mechanism and performance of TCGS. **a** Resistance changes within the strain range of 0 to 2%. **b** ΔR/R_0_-strain curves at 0.1%, 0.2%, 0.3%, 0.5%, 0.7%, 0.9%, and 1% strain. **c** ΔR/R_0_-strain curves under various applied frequencies at 0.3% strain. **d** Response and recovery times under 0.2% strain. **e** Long-term stability over 5000 cycles at 0.3% strain. **f** FEA simulations for models with $N$ of 0, 3, 5, and 7. **g** Displacement changes under different pressures for the model with $N$ = 7. **h** Stress distribution and current density modulus distribution within the micropore-crack synergistic structure under various strains. **i** Comparison of the sensitivity of flexible strain sensors recently reported in the literature with our sensor

The geometric properties of cracks, particularly depth and width, are crucial for the sensitivity of sensors. The opening and closing of cracks affect the conduction paths, resulting in significant changes in resistance, thereby enhancing strain detection sensitivity. The sensitivity of the strain sensor was quantified by the gauge factor, $\text{GF}=(\Delta R/{R}_{0})/\Delta \varepsilon$, where $\Delta R$ is the difference between instantaneous resistance ($R$) and initial resistance (${R}_{0}$), and $\Delta \varepsilon$ represents the applied strain [58]. In the testing of TCGS-7 with different crack widths, the results showed that the sensor with a width of 50 μm exhibited the highest sensitivity in the 0 to 2% strain range, achieving a maximum GF of 218.13, which is significantly higher than that of other widths (Figs. 2k, 3a, and S12). Narrow cracks have limited opening and closing ranges, resulting in minor changes in conductive paths and reducing sensitivity, while excessively wide cracks decrease conductive path variations, weakening mechanical strength and affecting stability. Regarding crack depth, as the crack depth of TCGS-7 increases from 65 to 155 μm, sensitivity initially increases and then decreases. The sensor with a depth of 130 μm exhibits the highest sensitivity at 2% strain, exceeding the sensitivities of the sensors with depths of 155 and 65 μm, which are 200.24 and 98.79, respectively (Figs. 2l, 3a, and S13). While deeper cracks enhance sensitivity, excessively deep cracks may reduce stability. Therefore, selecting a combination of a width of 50 μm and a depth of 130 μm achieves the best balance between sensitivity and stability for the sensor.

The number of turns ($N$) of the Archimedean spiral cracks was controlled by altering the metal mold’s geometry during μ-ECM without changing other geometrical factors, such as the crack depth and width. The electromechanical characteristics of the TCGS with Archimedean spiral cracks of varying $N$ were examined by measuring the change in relative resistance with strain. By comparing the sensitivities of TCGS-0, TCGS-3, TCGS-5, and TCGS-7 within the small strain range of 0 to 2%, it was found that the sensors’ sensitivity increased with the number of crack turns (Fig. S15). Specifically, the TCGS-7 exhibited a GF exceeding 97.35 in the strain range of 0 to 0.7%, and reaching 218.13 in the strain range of 0.7% to 2% (Fig. 3a), representing an approximately 4300% improvement compared to the maximum GF of 4.85 for the TCGS-0, which has no cracks. This result demonstrates the critical role of cracks in enhancing sensor sensitivity. Further tests were conducted to evaluate the detection limits of sensors with different $N$ through a series of comprehensive cyclic induction experiments in the ultra-low strain range of 0.1% to 1% (Figs. 3b and S16). The results showed that the detection limit decreased as the $N$ increased. The detection limit of TCGS-7 was as low as 0.1%, and even at this extremely low strain, the sensor could obtain reliable and distinguishable cyclic response signals, demonstrating its ability to accurately identify different strain levels. This exceptional precision in detecting subtle strain changes highlights the sensor’s potential for a variety of high-sensitivity applications. Additionally, by comparing TCGS-CM (GF of 1.49), TCGS-0 (GF of 4.85), and TCGS-M (GF of 14.56) within the range of 0 to 2%, we further demonstrate the dominant role of cracks in enhancing sensor sensitivity (Fig. S17). While the micropores in TCGS-0 contribute to a moderate sensitivity improvement compared to TCGS-CM, the presence of cracks in TCGS-M leads to a significantly higher GF. However, the synergistic combination of both micropores and cracks achieves even higher sensitivity, highlighting the importance of these two features working together to optimize sensor performance.

The speed of response-relaxation plays a pivotal role in determining the sensor’s responsiveness to external forces. This was investigated at 0.3% strain with various frequencies of 0.1, 0.3, 0.5, 0.7, 1, and 3 Hz (Fig. 3c). The consistently rapid response signals demonstrate the sensor’s high efficiency in adapting to strains across a wide range of frequencies. As shown in Fig. 3d, a rapid response time of 350 ms and a recovery time of 700 ms are realized at a straining speed of 1000 mm min^−1^. When the sensor is subjected to the same pressures in both the X and Y directions, the output resistance changes virtually identically (Fig. S18). Additionally, the TCGS-7 maintains remarkable stability and durability, enduring over 5000 cycles without significant degradation, even when subjected to an ultralow strain of just 0.3% at a frequency of 1 Hz (Fig. 3e). This proves the sensor’s dependability and suitability for long-term applications. Notably, the outstanding superhydrophobicity of the sensor ensures stable sensing performance even after 15 days of submersion in water (Fig. S19), making it ideal for applications in humid environments.

In order to better understand the sensing mechanism of the TCGS, finite element analysis (FEA) was utilized to analyze the micropore-crack synergistic effects. Initially, we constructed four distinct FEA models to simulate Archimedean spiral cracks with $N$ values of 0, 3, 5, and 7, each featuring a crack width of 0.2 mm and depth of 0.5 mm within a substrate size of 40 × 40 × 1 mm^3^ (Fig. 3f and Note S2). The results indicate that the presence of the cracks significantly reduces the mechanical strength of the material. A denser distribution of cracks is associated with an enhanced displacement response to external forces, thus showing increased sensitivity, as demonstrated in Fig. S15. The models with Archimedean spiral cracks exhibit consistent maximum displacements in both X and Y directions due to the unique continuous arc geometry of the cracks. The Von Mises stress is primarily concentrated at the base of the spiral cracks, and the continuous curvilinear shape of these spiral cracks optimizes the stress distribution, thereby reducing stress concentration in any single area. As $N$ increases, the crack distribution becomes more uniform, effectively reducing local stress concentrations and thus decreasing the maximum stress levels within the model (Fig. S20). This uniform distribution results in a more even stress distribution across the X and Y axes, thus minimizing the variance in maximum stress values between these two directions, ensuring consistent sensitivity and stable performance regardless of the direction of applied forces, as evidenced by Fig. S18.

Furthermore, the simulations with progressively increasing loads from 0.01 to 0.07 MPa along the X and Y axes demonstrated that both displacement and Von Mises stress in the model increased linearly with the load, confirming the model’s consistent responsiveness to load variations (Figs. 3g and S21–S24). Owing to their unique geometric shape, these Archimedean spiral cracks serve as the paths of least resistance, facilitating stress propagation and leading to displacement radiating outward from the center of the model. With an increasing $N$, each turn of the cracks diminishes the intensity of the stress wave passing through, significantly reducing stress concentration at the model’s center, thus enhancing its structural integrity and stability. In contrast, the model devoid of cracks ($N$ = 0) experiences displacement from the ends towards the center, attributable to a homogeneous stress distribution, leading to increased end displacement as pressure rises. Consequently, the Archimedean spiral cracks play a key role in enhancing the sensitivity of the sensor. This enhancement results from a marked resistance change due to increased strain emanating from the center outward, which provides a solid foundation for the enhancement of the sensitivity of the strain sensors.

To further investigate the synergistic effects of the cracks and micropores in strain sensors, a comprehensive model integrating both features was developed (Fig. 3h and Note S3). As the strain increases, the Von Mises stress significantly rises, particularly at 3% and 5% strain, where high-stress areas become prominent and spread throughout the model (Fig. S25). Additionally, the increase in strain significantly affects the distribution of current density. With no strain, the current primarily flows along paths with lower resistance between the micropores. As the strain increases, the deformation of the micropores and cracks alters the conductive pathways, causing high current density areas to disperse and expand, thereby increasing the overall resistance of the material. As discontinuous elements, the micropores play a crucial role in preventing rapid crack propagation and reducing stress concentration at crack locations, effectively preventing the formation of large-scale stress concentration areas. Additionally, micropores increase high current density areas, significantly enhancing the rate of resistance change. Therefore, the synergistic effects of cracks and micropores greatly reduce the risk of material failure, enhance the detection of minute strain changes, and improve the sensitivity and stability of the sensor. By adjusting the size, distribution, and location of micropores and cracks, the sensors can be tailored for specific purposes. This innovative approach significantly surpasses conventional designs (Fig. 3i), paving the way for the creation of strain sensors with high sensitivity and stability.

### Wettability and Droplet Detection Performance

**Fig. 4:**
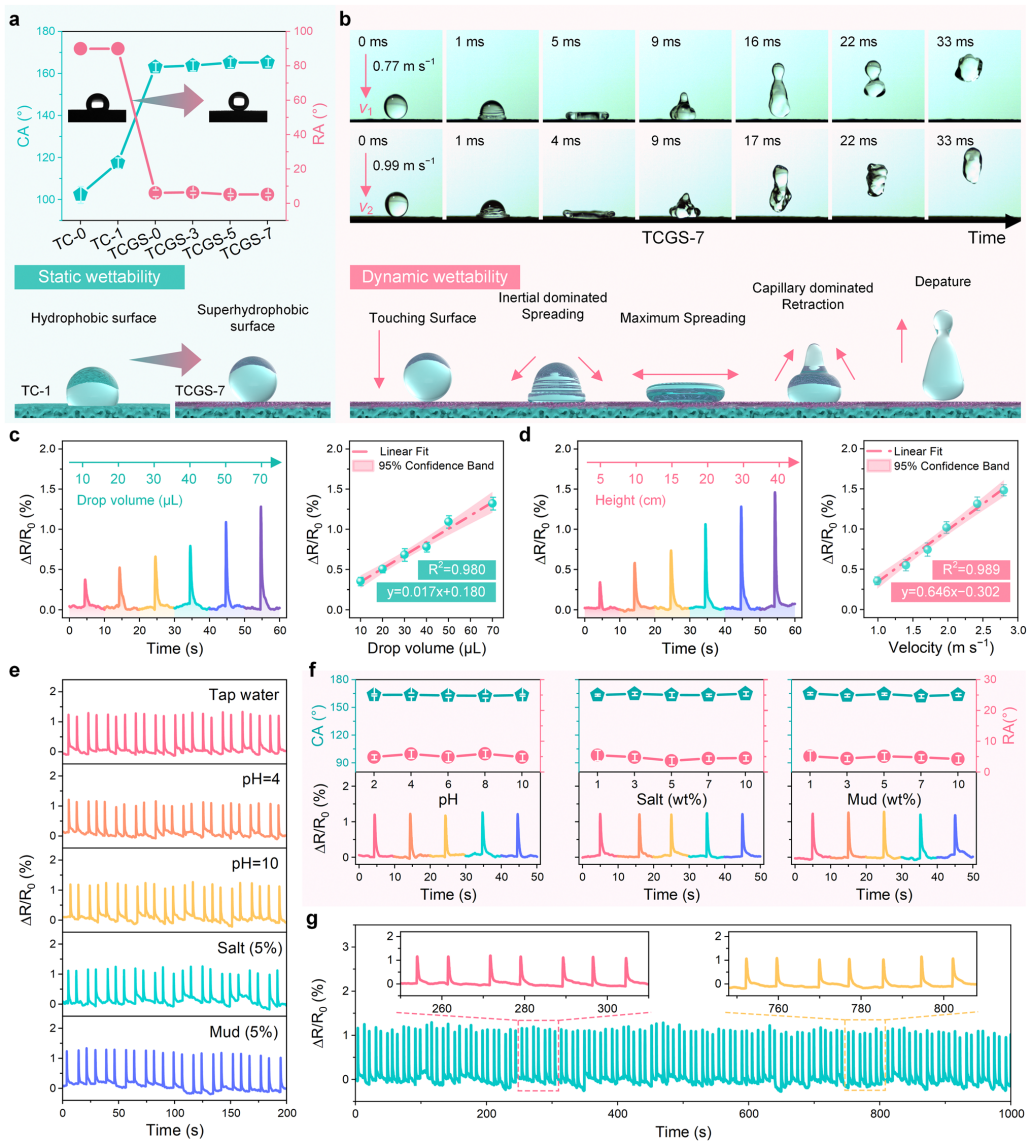
Analysis of sensor properties including static wettability, dynamic wettability, and electric response signals for various liquid droplets. **a** Analysis of CA, RA, and static wettability. **b** Selected snapshots showing droplets impacting the TCGS-7 surface at velocities of 0.77 and 0.99 m s^−1^, incorporating dynamic wettability analysis. **c** Electrical responses to water droplets of different sizes dropped from a height of 30 cm, including linear analysis. **d** Electrical responses to 70 μL water droplets falling from varied heights, with linear analysis. **e** Response to various compositions of water droplets: tap water, acidic (pH = 4), alkaline (pH = 10), 5% saline, and 5% mud solutions. **f** Analysis of CA, RA, and the electrical responses of droplets in solutions with varying pH levels, salt concentrations, and mud contents. **g** Long-term detection of electrical response signals from water droplets

Superhydrophobic surfaces are pivotal in enhancing the sensitivity and durability of sensors for liquid leakage detection due to their exceptional water repellency. This repellency is achieved through a synergistic combination of high surface roughness and low surface energy [34]. In this work, a cost-effective and scalable approach was employed to develop superhydrophobic surfaces on the sensors. First, GNS and PDMS were uniformly dispersed in an EA solution via ultrasonication. By precisely controlling the spraying speed, a balance between the rates of spraying and solvent evaporation was achieved, forming a uniform micro-nanostructured GNS/PDMS coating on the sensor’s surface. Upon heating, the PDMS cured and the TPU matrix softened, embedding the GNS firmly within the composite matrix, thereby ensuring the stability of the micro-nanostructures. As illustrated in Fig. 4a, the original surface (TC-0) had a contact angle (CA) of 102 ± 4° and a rolling angle (RA) of 90°. After coating (TCGS-7), the CA increased to 165 ± 2°, and the RA significantly decreased to 5 ± 1°, suggesting enhanced surface roughness and improved droplet mobility. The transition from a hydrophobic to a superhydrophobic state is attributed to the combined effects of the increased roughness and the low surface energies of the GNS/PDMS coating [61]. According to the Cassie-Baxter model, these surface micro-nanostructures are capable of trapping large amounts of air, which effectively reduces the contact area of the droplets with the surface, thereby drastically improving the contact angle and achieving superior superhydrophobicity [33]. Moreover, even after over 300 cycles of bending and stretching, TCGS-7 retained a CA above 150° and a RA below 10° (Fig. S26). These results confirm that TCGS-7 can effectively maintain its superhydrophobic characteristics under significant mechanical stress, demonstrating its robustness and suitability for applications involving frequent bending and stretching.

The dynamic wettability of the superhydrophobic surface was further analyzed to assess its applicability in the real world. In experiments, water droplets of 10 μL were released onto the surface from varying heights (3, 5, 7, and 10 cm). Utilizing the free-fall formula ($v=\sqrt{2gh}$), the impact velocities ($v$) of water droplets on the TCGS-7 surface were calculated to be 0.77, 0.99, 1.17, and 1.40 m s^−1^, respectively. The dynamic processes were captured using a high-speed video camera at 10,000 fps (Videos S1–S4), and typical snapshots are shown in Figs. 4b and S27. Initially, the droplet undergoes inertial dominated spreading upon first contact with the superhydrophobic surface, characterized by a decrease in height [62]. As spreading continues, the morphology of the droplet evolves from a spherical shape to a cap shape and then to a disk shape, simultaneously experiencing a decrease in kinetic energy that leads to an increase in surface energy. At maximum spread, the surface energy peaks while the kinetic energy nears depletion. This is followed by a retraction process dominated by surface tension, which draws the droplet rim towards the center and increases its height, thereby leading to a decrease in surface energy and an increase in kinetic energy. The spreading length decreases until it reaches zero, at which point the droplet rebounds. Due to energy dissipation during surface contact, the kinetic energy of the rebound is lower than that of the initial impact. Similarly, the TCGS-7 demonstrates its robust resistance to water jets, as a jet at 7.5 m s^−1^ is completely rebounded without leaving residue on the TCGS surface (Fig. S28). Throughout the impact process, the water droplets and water jets remain superhydrophobic in various states, proving that the TCGS-7 surface has excellent resistance to water impact.

TCGS-7 has excellent cutting properties, allowing for the fabrication of samples in various shapes tailored to different application scenarios, with sensitivity varying based on the specific shape. This variation in sensitivity primarily arises from differences in the sensor’s micropores and crack structures, which significantly affect its performance. To further evaluate the sensor’s ability to detect water droplets, a strip sensor (5 × 40 × 1 mm^3^) was prepared for testing. The TCGS-7 strip sensor can withstand strains of up to 5% and exhibits a marked increase in sensitivity, reaching a GF of 991.62 (Fig. S29). The sensor was fixed in a cantilever configuration to detect the response signal from water droplets, as shown in Fig. S30. Different volumes of water droplets (10, 20, 30, 40, 50, and 70 μL) were precisely controlled using injection needles with varying inner diameters (Fig. S31). The droplets were dropped from a height of 30 cm above the sensor, producing single-peak response signals with varying amplitudes (Fig. 4c). Although all droplets contact the sensor at the same impact velocity (2.42 m s^−1^) due to free fall, their different volumes lead to changes in the impact force. The impact force is determined by the formula:

1 $$F = \frac{\Delta P}{{\Delta t}} = \frac{{m\left( {v_{f} - v_{i} } \right)}}{\Delta t}$$

where $m$ represents the mass of the droplets, ${v}_{f}$ and ${v}_{i}$ are the velocities of the droplets after and before impact, respectively, and $\Delta t$ is the contact time. Assuming ${v}_{f}=0$ (If the velocity of water droplets after impact decreases to zero, or the rebound velocity can be ignored), the formula simplifies to

2 $$F = \frac{{mv_{i} }}{\Delta t}$$

The deformation (deflection) of the cantilever sensor is derived using the theory of bending in material mechanics [63]. The maximum deflection is calculated with:

3 $$\Delta = \frac{{FL^{3} }}{3EI}$$

where $F$ is the force applied on the sensor, $L$ is the length of the sensor, $E$ is the elastic modulus of the material, and $I$ is the moment of inertia of the sensor section. Integrating these principles, the strain ($\varepsilon$) in the sensor is expressed as:

4 $$\varepsilon = \frac{\Delta L}{L} = \frac{{\rho V\sqrt {2gh} L^{2} }}{3EI\Delta t}$$

This formulation demonstrates that the strain is directly proportional to both the drop height and droplet volume, and also depends on the physical and structural properties of the sensor. Changes in droplet height and droplet volume directly influence the deformation degree of the sensor. These deformations alter the cracks and micropores of the sensor, affecting both the GNS conductive network on its surface and the CNTs network inside, thus resulting in changes in resistance. By establishing a linear correlation between the resistance changes and droplet volume, the following formula is derived:

5 $$y = 0.017x + 0.180\;(R^{2} = 0.980)$$

This confirms a strong linear relationship between the droplet volume and the signal change magnitude, highlighting the sensor’s high precision in detecting water droplets of varying sizes.

To investigate the effect of impact velocities on the deformation of the sensor, experiments involving water droplet impact at varying velocities were conducted (Fig. 4d). By adjusting the falling height, the impact velocities of 70 μL water droplets were calculated to be 0.99, 1.40, 1.71, 1.98, 2.42, and 2.8 m s^−1^, respectively. As the impact velocity increases, the corresponding impact force also increases, resulting in greater deformation of the sensor. This increased deformation exacerbates the discontinuity between the GNS and CNTs conductive network, thereby leading to amplified changes in resistance. A linear relationship between the impact velocities of water droplets and the peak changes in resistance signals was established, as evidenced by the equation:

6 $$y = 0.646x - 0.302\;\left( {R^{2} = 0.989} \right)$$

This exhibits a strong linear relationship between the impact velocity and the signal change amplitude, indicating the sensor’s consistent and predictable response to mechanical stress. The sensor’s exceptional performance stems from the synergistic interaction between the micropores and cracks, which rapidly induce resistance changes by adjusting the conductive network of GNS and CNTs. Additionally, the sensor’s superhydrophobic surface plays a crucial role not only in enhancing durability against repeated water impacts but also in improving sensitivity by minimizing baseline noise caused by water adhesion. These capabilities enable the sensor to sensitively monitor water droplet response signals and estimate the droplet size and impact velocity, making it suitable for droplet detection under various conditions.

Furthermore, the electrical response of the sensor to leakage from various liquids was tested. The sensor exhibited consistent and reliable response behavior with different liquids, including tap water, solutions with different pH values, salt solutions ranging from 1% to 10% concentration, and mud with 1% to 10% concentration (Figs. 4e and S32). On the sensor surface, different droplets all exhibit large CA > 160° and small RA < 10° (Fig. 4f). Different droplets (70 µL) dropped from a height of 30 cm produce significant signals, regardless of the droplet composition. Further testing of the sensor’s long-term leakage detection capability revealed that it possessed long-lasting stability, indicating that it is suitable for long-term droplet leakage detection (Fig. 4g). This resistance to acid, alkali, salt, and mud is credited to the material and structural design of the sensor, particularly the TPU base and its stable GNS/PDMS superhydrophobic coating. In summary, the remarkable sensitivity and stability of the sensor in a range of liquid environments make it exceptionally suitable for leakage detection applications.

### Applications of TCGS in Liquid Leakage Detection

**Fig. 5:**
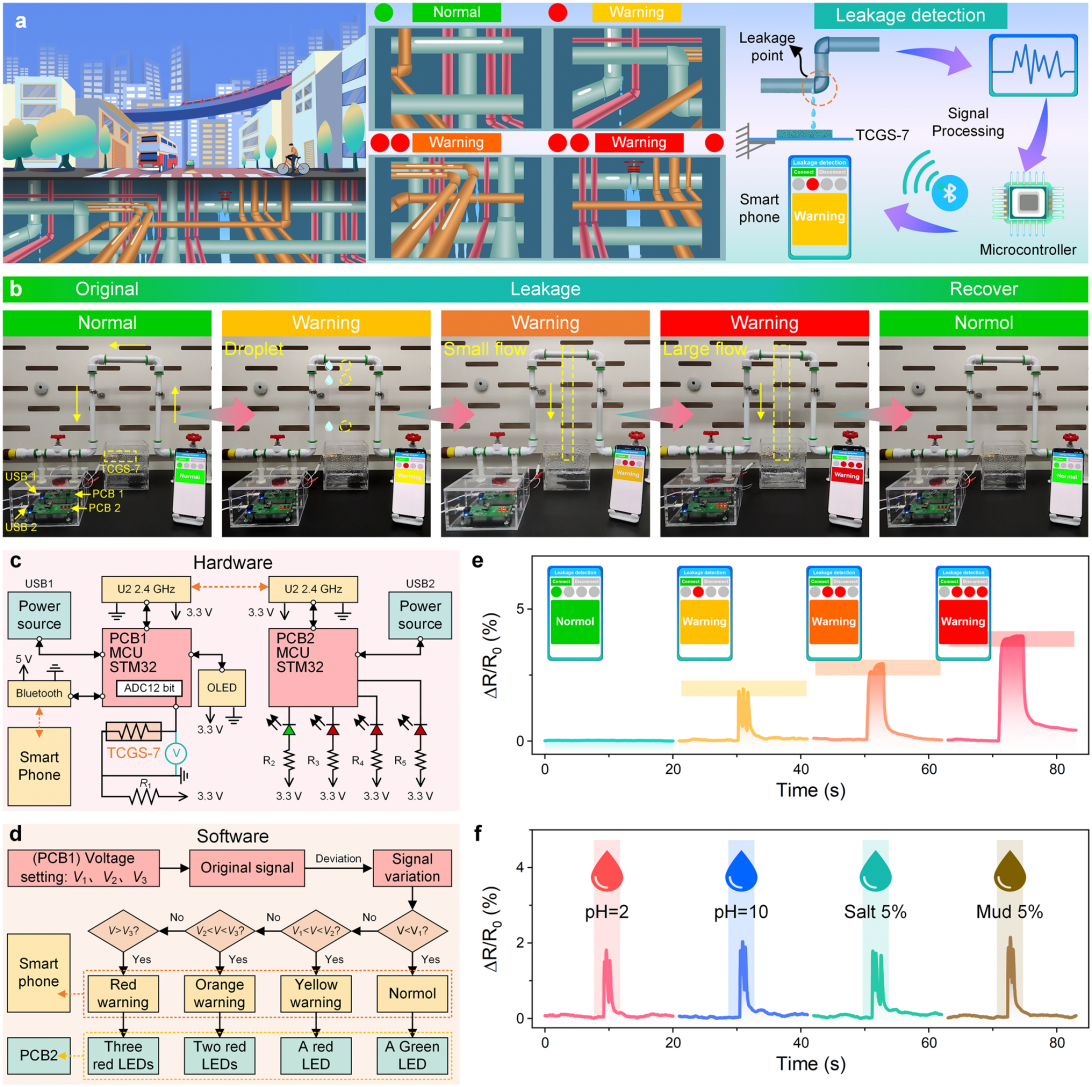
Applications of TCGS-based leakage detection device. **a** Overview of pipe distribution and leakage beneath the city (left), an enlarged view of different pipe leakages (center), and the components of TCGS-based leakage detection device (right). **b** Liquid leakage detection process: from a normal state, to water droplet leakage, small water flow leakage, large water flow leakage, and finally, to recovery. **c** Design of the hardware for leakage detection. **d** Design of the software. **e** Response signals for normal state, droplet leakage, small flow leakage, large flow leakage. **f** Leakage detection for various liquids: acidic (pH = 5), alkaline (pH = 10), saline (5% concentration), and mud (5% concentration)

Underneath cities, the complex networks of pipelines serve as the lifeline of urban operations, transporting water and other essential liquids. However, frequent leakages not only waste resources but also pose environmental and public safety risks. In response to this challenge, the use of the TCGS for leakage detection offers an effective solution. Specifically, the electrodes of the TCGS are connected to a microcontroller, which captures changes in resistance signals caused by liquid leakages and wirelessly transmits these signals to a smartphone via Bluetooth or WiFi, enabling remote monitoring (Fig. 5a). Additionally, the leakage detection device visually indicates the severity of leakages through warnings of different colors, ranging from green for no leakage to red for serious leakage, thus enabling pipeline managers to quickly identify and address these issues. The widespread deployment of these leakage detection devices in urban pipeline systems ensures that the leakages can be detected and located promptly, even in areas that are hidden or difficult to access.

In practical application, an experimental scenario was established to simulate pipeline leakages (Fig. 5b). By controlling the size of the leakage point and the flow rate, various situations of droplet leakage, small water flow leakage, and large flow leakage were successfully simulated. In this scenario, the TCGS-7 was fixed on a container in the form of a cantilever beam, and its electrodes were connected to an STM32 chip on PCB1 (Fig. S33). The TCGS-7 was connected in parallel with a resistor ${R}_{1}$, and the voltage change in the sensor was monitored through an ADC module of the STM32 chip to determine the change in resistance (Fig. 5c). The signal was wirelessly transmitted to the STM32 chip on PCB2, which converted the signal into a visual LED alarm signal (Fig. S34). At the same time, the signal was also sent to a smartphone via Bluetooth to display alerts of different levels. By programming specific parameters, voltage values corresponding to different degrees of liquid leakages could be set (Fig. 5d). For example, a normal state corresponds to voltage ${V}_{1}$, droplet leakage to voltage ${V}_{2}$, small flow leakage to voltage ${V}_{3}$, and large flow leakage to voltage ${V}_{4}$. When the voltage exceeds the preset value, the LEDs light up, and the corresponding alarm signal is displayed on the smartphone. Specifically, in the scenario shown in Fig. 5b, e and Videos S5–S9, when there is no leakage, the sensor remains undeformed, and a green LED is lit, with the phone displaying a green “Normal”. When a droplet leakage occurs, the sensor deforms slightly, and a red LED is lit, with the phone displays a yellow “Warning”. A small flow leakage causes a more significant deformation of the sensor, lighting up two red LEDs, with the phone displaying an orange “Warning”. When a large flow leakage occurs, the sensor deforms even more significantly, and three red LEDs light up, with the phone displaying a red “Warning”. After the leakage is repaired and there is no more liquid leakage, the sensor’s resistance value returns to normal, a green LED is lit, and the phone displays a green “Normal”, indicating that the system has returned to its original state. These differences enable the leakage detection device to effectively differentiate between different degrees of liquid leakages, providing extremely reliable information for the timely repair of the leakage points.

For various compositions of liquid leakages, including acidic (pH = 5), alkaline (pH = 10), saline (5% concentration), and mud (5% concentration), the leakage detection device also demonstrates high sensitivity in detecting droplet leakage, small flow leakage, and large flow leakage (Figs. 5f and S35–S38). This finding indicates that the leakage detection device is not only capable of detecting leakages from different liquids but also effectively assesses the severity of various liquids leakages. Therefore, the TCGS-based leakage detection device demonstrates wide applicability and efficiency in a variety of practical application scenarios, providing critical early warnings for both industrial pipelines and potential liquid leakage situations in daily life, thereby offering valuable time windows to prevent potential damage. This advancement is expected to significantly improve the efficiency of urban pipeline management, reduce the risk of leakages, and make a crucial contribution to the sustainable use of water resources and the development of a safe and efficient urban environment.

## Conclusion

In summary, we have successfully developed a TCGS leakage detection device characterized by exceptional sensitivity and stability, capable of sensitively detecting and providing warnings for liquid leakages with high precision. Inspired by the sensing mechanisms of scorpions, the TCGS utilizes the synergistic effects of micropores and Archimedean spiral cracks, displaying the following advantages: (1) the manufacturing method combines μ-ECM and surface modification, offering high cost-effectiveness, simplicity, and scalability; (2) the Archimedean spiral crack arrays enhance the sensor’s sensitivity by 4300%, achieving a sensitivity of 218.13 at a strain of just 2%; (3) the synergistic structure of micropores and cracks significantly reduces stress concentration, greatly improving the sensor’s stability and ensuring a lifespan of over 5000 cycles; (4) the robust superhydrophobicity of the TCGS effectively prevents liquid adhesion and maintaining high sensitivity and stability even in wet environments; (5) it is capable of quantitatively detecting small volumes of liquid at various sizes and velocities, with a linear response exceeding 0.98; (6) the device supports real-time wireless detection of leakages from a variety of liquids, including water, acids, alkalis, saline solution, and mud. The TCGS-based device covers a wide range of leakage scenarios from droplets to large flow leakages and can issue timely alarms. Therefore, the detection device exhibits practical value in preventing global liquid leakage and promoting sustainable development, owing to its exceptional environmental adaptability and scalability.

## Supplementary Information

Below is the link to the electronic supplementary material.

Supplementary file1 (DOCX 30704 KB)

Supplementary file2 (MP4 7828 KB)

Supplementary file3 (MP4 7764 KB)

Supplementary file4 (MP4 7798 KB)

Supplementary file5 (MP4 7774 KB)

Supplementary file6 (MP4 11361 KB)

Supplementary file7 (MP4 10937 KB)

Supplementary file8 (MP4 10199 KB)

Supplementary file9 (MP4 10696 KB)

Supplementary file10 (MP4 9810 KB)
