# Supplementary material for: Bioinspired Ultrasensitive Flexible Strain Sensors for Real-Time Wireless Detection of Liquid Leakage
Source: Nanomicro Lett. 2024 Nov 22;17:68. doi: 10.1007/s40820-024-01575-2 (PMC11582251; doi:10.1007/s40820-024-01575-2)
Supplement: Supplementary file 1 — Supplementary file1 (DOCX 30704 KB) [file 40820_2024_1575_MOESM1_ESM.docx]

Supporting Information for

**Bioinspired Ultrasensitive Flexible Strain Sensors for Real-Time Wireless Detection of Liquid Leakage**

Weilong Zhou^1^, Yu Du^1^, Yingying Chen^1^, Congyuan Zhang^1^, Xiaowei Ning^1^, Heng Xie^2,^*, Ting Wu^1,^*, Jinlian Hu^3,^*, Jinping Qu^1^

^1^Key Laboratory of Material Chemistry for Energy Conversion and Storage, Ministry of Education; Hubei Key Laboratory of Material Chemistry and Service Failure and Hubei Engineering Research Center for Biomaterials and Medical Protective Materials; School of Chemistry and Chemical Engineering, Huazhong University of Science and Technology, Wuhan 430074, P. R. China

^2^School of Materials Science and Engineering, Wuhan Institute of Technology, Wuhan 430205, P. R. China

^3^Department of Biomedical Engineering, City University of Hong Kong, Hong Kong, Hong Kong SAR 999077, P. R. China

*Corresponding authors. E-mail: [hengxie@hust.edu.cn](mailto:hengxie@hust.edu.cn) (Heng Xie), [tingwu@hust.edu.cn](mailto:tingwu@hust.edu.cn) (Ting Wu), [jinliahu@cityu.edu.hk](mailto:jinliahu@cityu.edu.hk) (Jinlian Hu)

## Supplementary Notes

Note S1 **Structural principle and dispersion mechanism of ERE**

The mechanism behind the enhanced mixing and dispersion of Na_2_SO_4_, CNTs, and TPU in the eccentric rotor extruder (ERE) is detailed here. As shown in Fig. S3a, the ERE used in this study consists of a stator with an inner diameter of 40 mm and rotors with a diameter of 33.5 mm, an eccentricity of 3 mm, and a rotational speed of 35 r/min. Unlike traditional extruders, the ERE’s design emphasizes the complex dynamic interaction between the stator and the dual eccentric rotors, which significantly improves material mixing and dispersion. The rotors consist of alternating spiral and straight segments, rotating and revolving in opposite directions within the intersecting cavities of the stator (Fig. S3b). This unique setup creates alternating converging and diverging flows, resulting in an intense elongational flow.

As shown in Fig. S3c, the cavity volume changes periodically in a sinusoidal pattern, alternately expanding and contracting. This periodic change causes alternating converging and diverging flows of the melt, which is crucial for processing the materials. In the converging regions, as the cavity volume decreases, the polymer particles are compressed and stretched into thin flakes with increased surface area. Upon entering the diverging regions, the rapid release of pressure causes these flakes to break into smaller fragments due to the sudden expansion of the melt. This cyclic process of compression and expansion effectively disperses agglomerated fillers, such as Na_2_SO_4_ and CNTs, ensuring their uniform distribution throughout the TPU melt. The elongational flow field also aligns and disperses fillers along the flow direction. As the melt enters the diverging regions, the stretching forces continue to break down filler aggregates, allowing the melt to encapsulate and distribute them uniformly. This mechanism not only optimizes the dispersion of Na_2_SO_4_ and CNTs in the TPU matrix but also ensures uniform mixing, significantly enhancing the overall properties of the final materials.

### Note S2 Finite Element Analysis (FEA) of the bioinspired models with $\boldsymbol{N}$ of 0, 3, 5, and 7

The bioinspired models were built for FEA simulations by mimicking the geometry of the Archimedean spirals with various numbers of turns ($N$) of 0, 3, 5, and 7 (Fig. S11). Each model features a crack with a width of 0.2 mm and a depth of 0.5 mm, set within a substrate size of 40 mm × 40 mm × 1 mm. The software used was Workbench (2023 R1). The sensors were fabricated from flexible materials, characterized by a Young’s modulus of 2.727 MPa, a density of 0.586 g cm^−3^, and a Poisson’s ratio of 0.36. During the simulation, pressures of 0.01 MPa were applied to both ends of the model along the X and Y axes, simulating the displacement and Von mises stress distributions for the molds with varying $N$ of 0, 3, 5, and 7, respectively (Figs. 3f and S20). The results indicated that the presence of cracks significantly reduced the mechanical strength of the material, particularly in the regions with dense cracks, leading to substantial increases in displacement under the consistent loads. As $N$ increased, the crack distribution became more uniform, effectively reducing local stress concentrations and thereby lowering the maximum stress levels within the model. This adjustment resulted in a more uniform stress distribution across the X and Y axes, thereby minimizing the variance in maximum stress values between these two directions.

Furthermore, simulations with progressively increasing loads from 0.01 to 0.07 MPa along the X and Y axes demonstrated that both displacement and Von mise stress in the model increased linearly with the load, confirming the model’s consistent responsiveness to load variations (Figs. 3g and S21–S24). Stress was primarily concentrated at the base of the spiral cracks, and the continuous curvilinear shape of these spiral cracks optimized the stress distribution, reducing stress concentration in any single area. With an increasing $N$, each turn of the crack diminished the intensity of the stress wave passing through, significantly reducing stress concentration at the model’s center, thus enhancing its structural integrity and stability.

### Note S3 FEA of the micropore-crack synergistic structure

To further investigate the synergistic effects of the micropores and cracks in strain sensors, we constructed a bioinspired model representing the cross-section of the Archimedean spiral crack model for FEA (Figs. 3h and S25). The model features a crack with a width of 0.1mm and a depth of 0.5mm, filled with micropores. The software used was COMSOL Multiphysics 6.0. The material properties of the model are as follows: a Young’s modulus of 2.727 MPa, a density of 0.586g cm^−3^, a Poisson’s ratio of 0.36, and an electrical conductivity of 9.019 × 10^−4^. During the simulation, a voltage of 1 V is applied at one end of the model, with the other end grounded, to study the distribution of stress and current density under strains of 0%, 1%, 3%, and 5%. As the strain increases, the Von Mises stress significantly rises, particularly at 3% and 5% strain, where high-stress areas become more prominent and spread throughout the model. Additionally, the increase in strain affects the distribution of current density. When there is no strain, the current predominantly follows paths of lower resistance between the micropores. As the strain increases, the deformation of the micropores and cracks results in a dispersed area of high current density. This dispersion demonstrates that the deformations of micropores and cracks substantially alter the conductive pathways within the material, leading to an overall increase in electrical resistance. As discontinuous elements, the micropores play a crucial role in preventing rapid crack propagation and reducing stress concentration at crack locations, effectively preventing the formation of large-scale stress concentration areas. Additionally, micropores also lead to an increase in high current density areas, thereby significantly increasing the rate of resistance change.

## Supplementary Figures


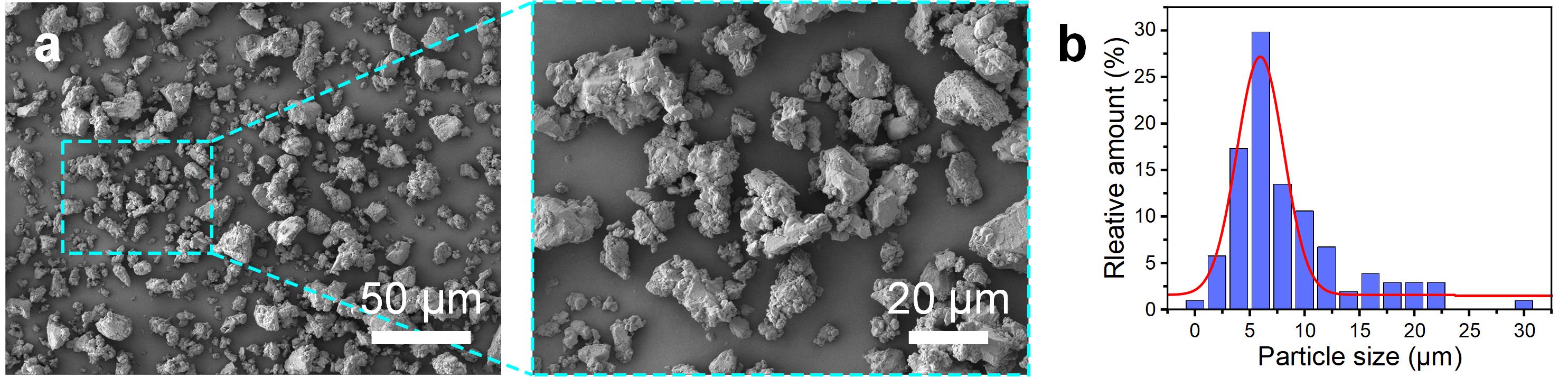


Fig. S1 **a** Micromorphology and **b** particle size distribution of Na_2_SO_4_


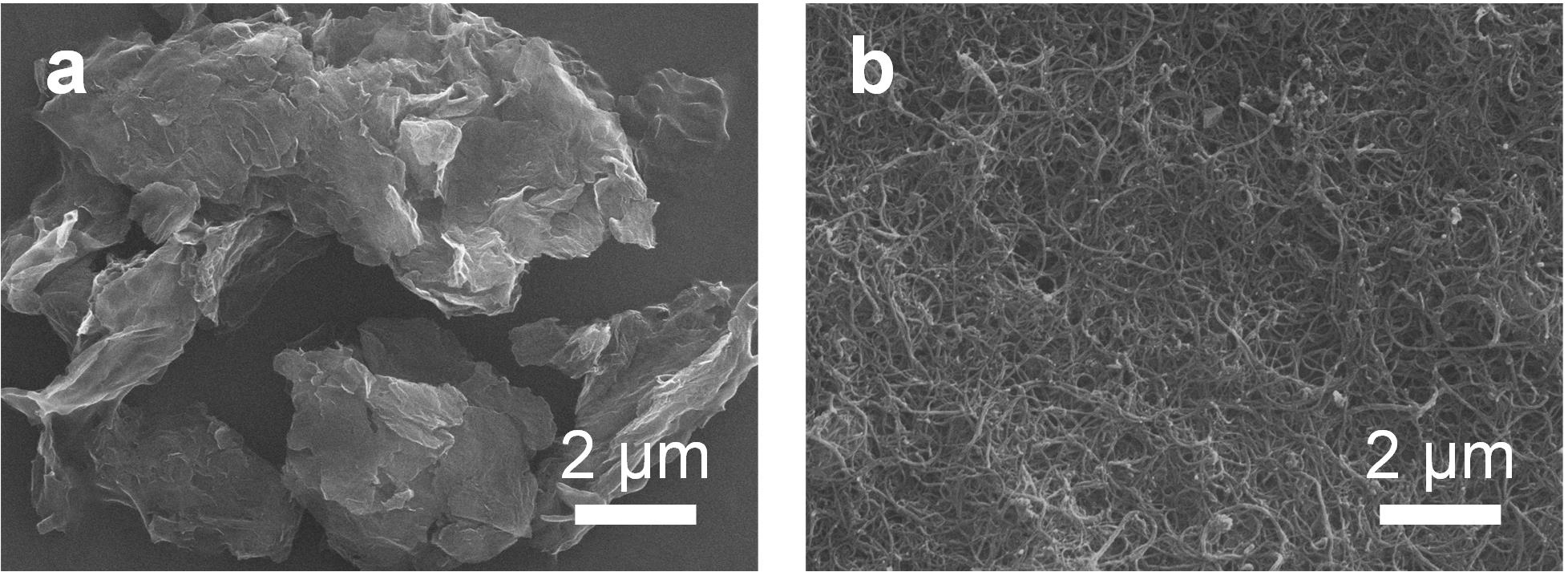


Fig. S2 Scanning electron microscope (SEM) images of **a** GNS and **b** CNTs


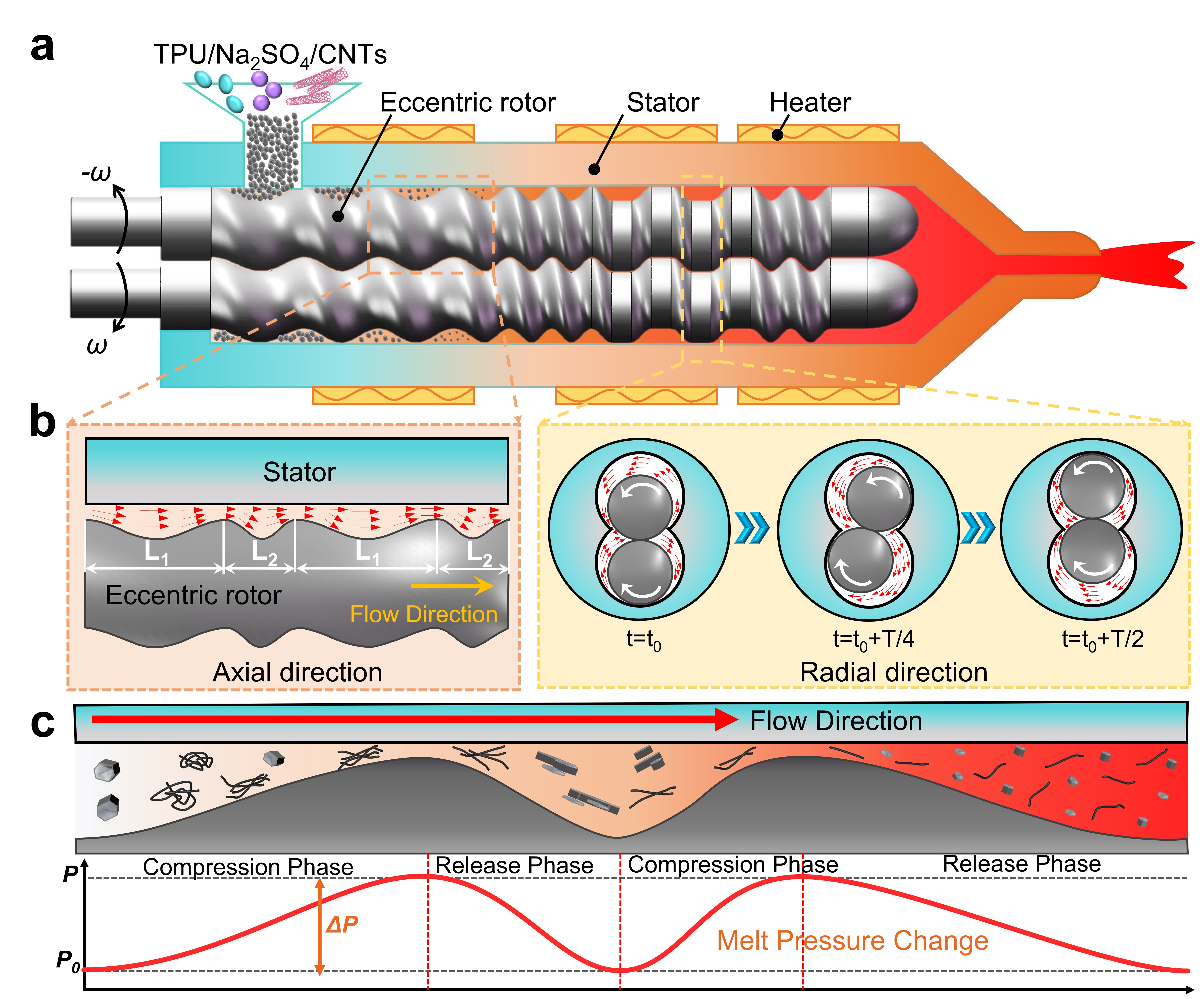


Fig. S3 Operating mechanism of the eccentric rotor extruder (ERE). **a** Schematic diagram of the ERE. **b** Changes in the ERE’s cavity volume throughout a rotational cycle. **c** Diagram of mixing and dispersing mechanism driven by a volumetric extensional flow field


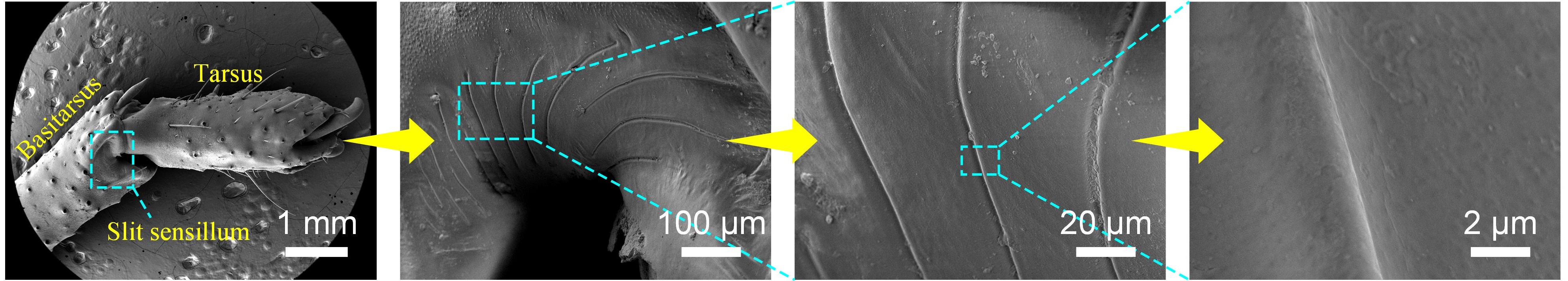


Fig. S4 Micromorphology of the scorpion crack sensillum

The microstructure of the crack sensillum of a mature forest scorpion (*Heterometrus petersii*) is depicted. SEM images indicate that the sensillum comprises a dozen curved cracks. In this work, the crack structure is imitated.


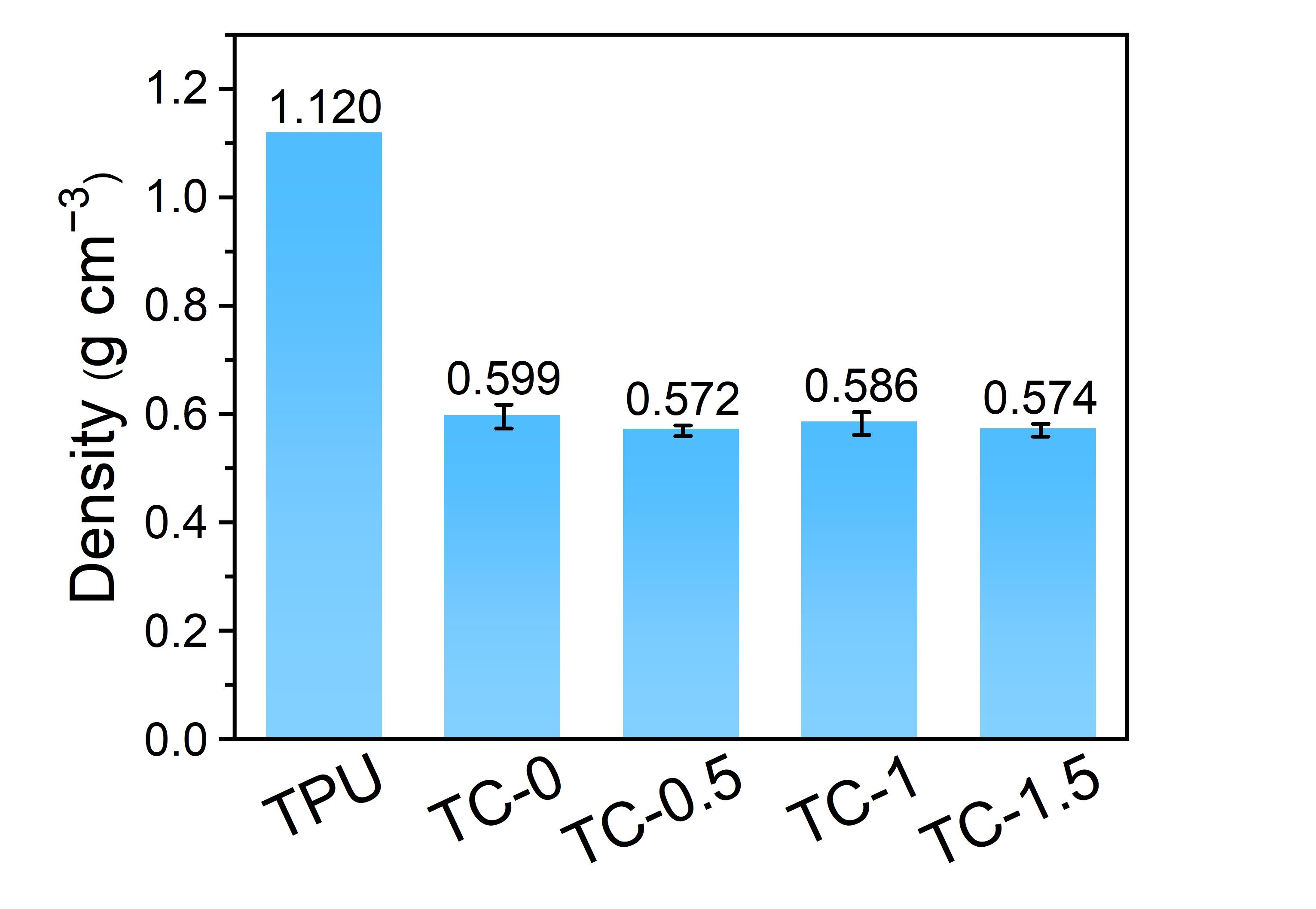


Fig. S5 Density comparison of TPU and TC foams


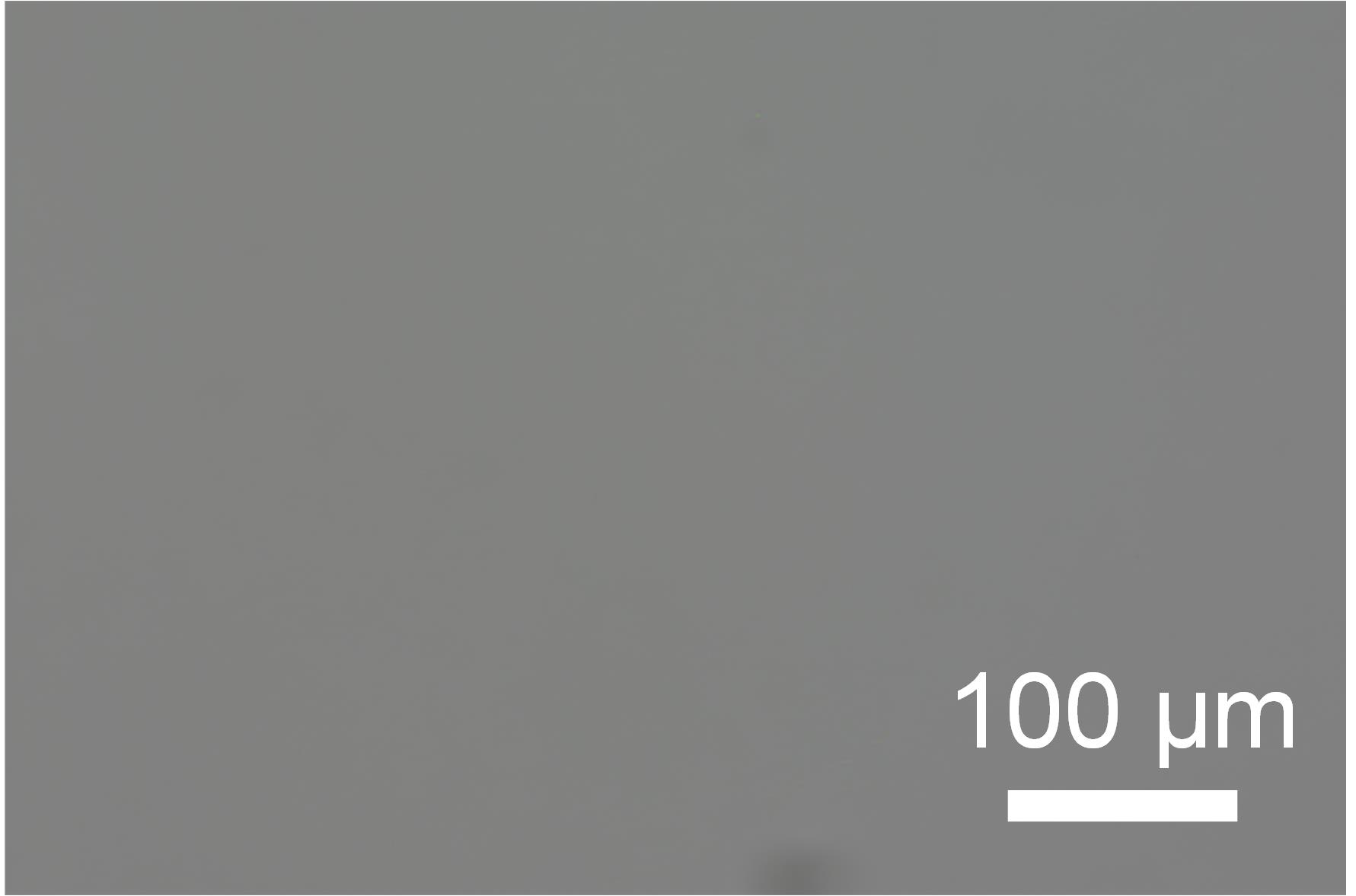


Fig. S6 Microscopic image of the solution after immersing TC-1


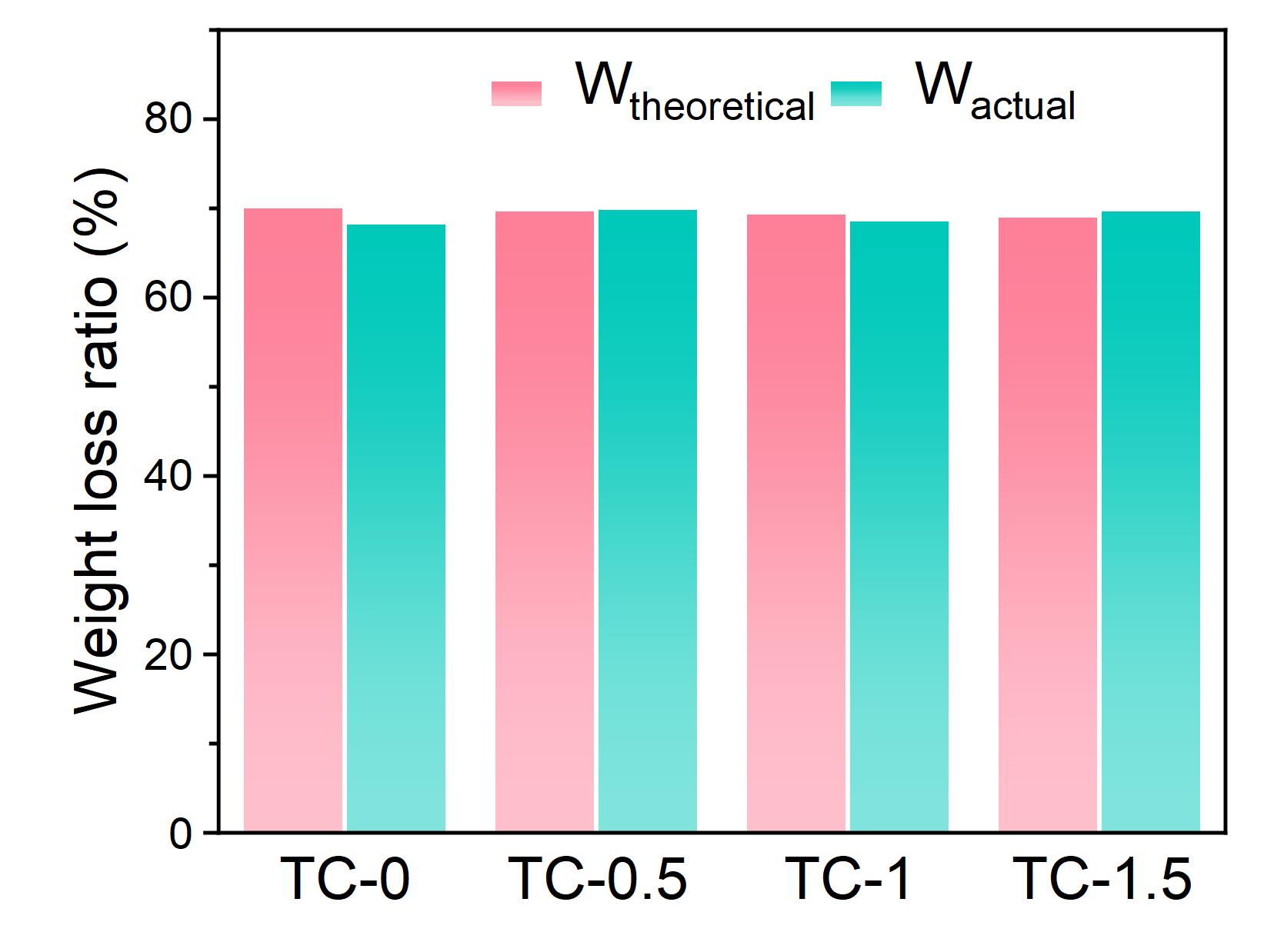


Fig. S7 The theoretical and actual weight loss ratio of TPU/CNTs/Na_2_SO_4_ composite after extraction of Na_2_SO_4_


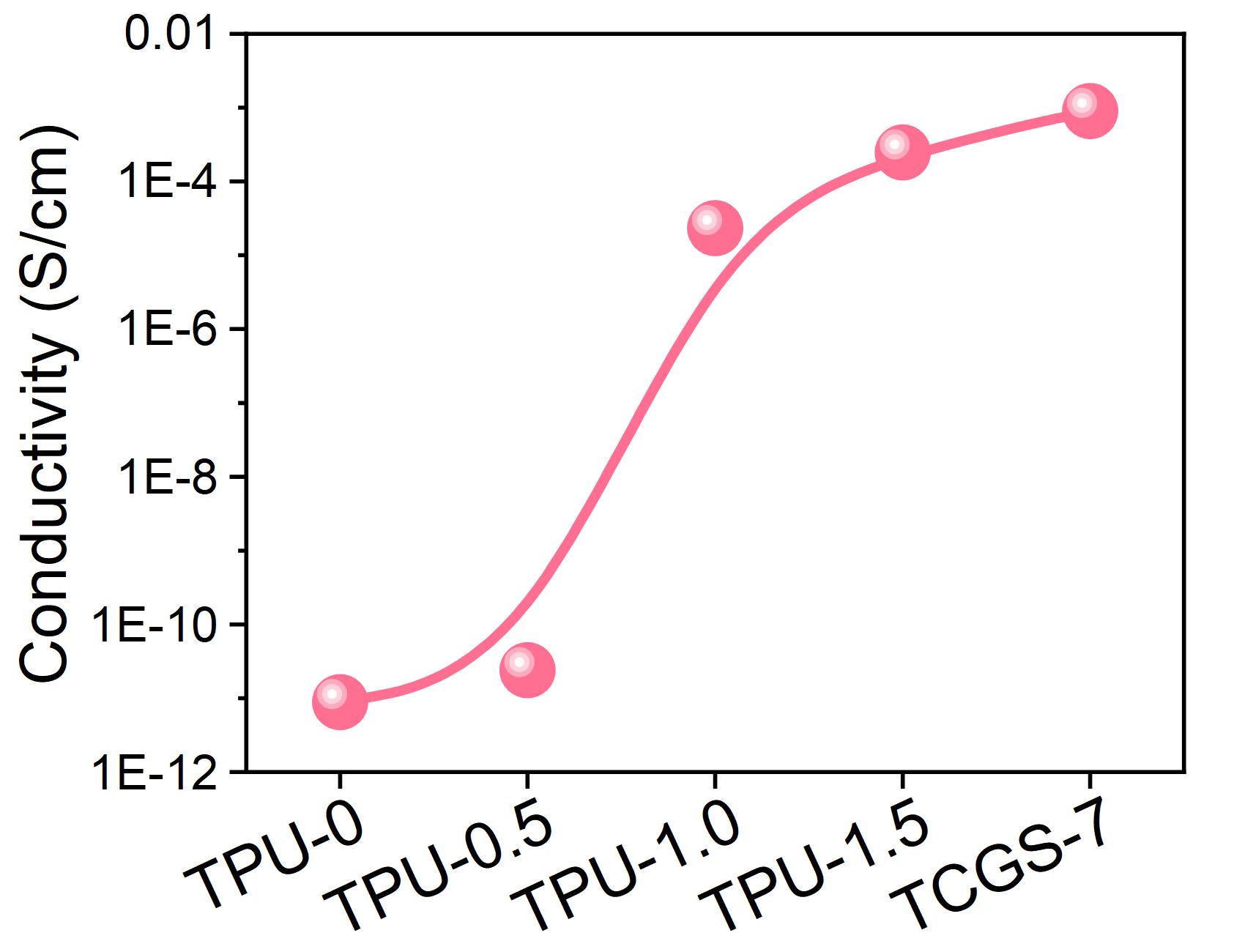


Fig. S8 Electrical conductivity of TC-0, TC-0.5, TC-1, TC-1.5, and TCGS-7


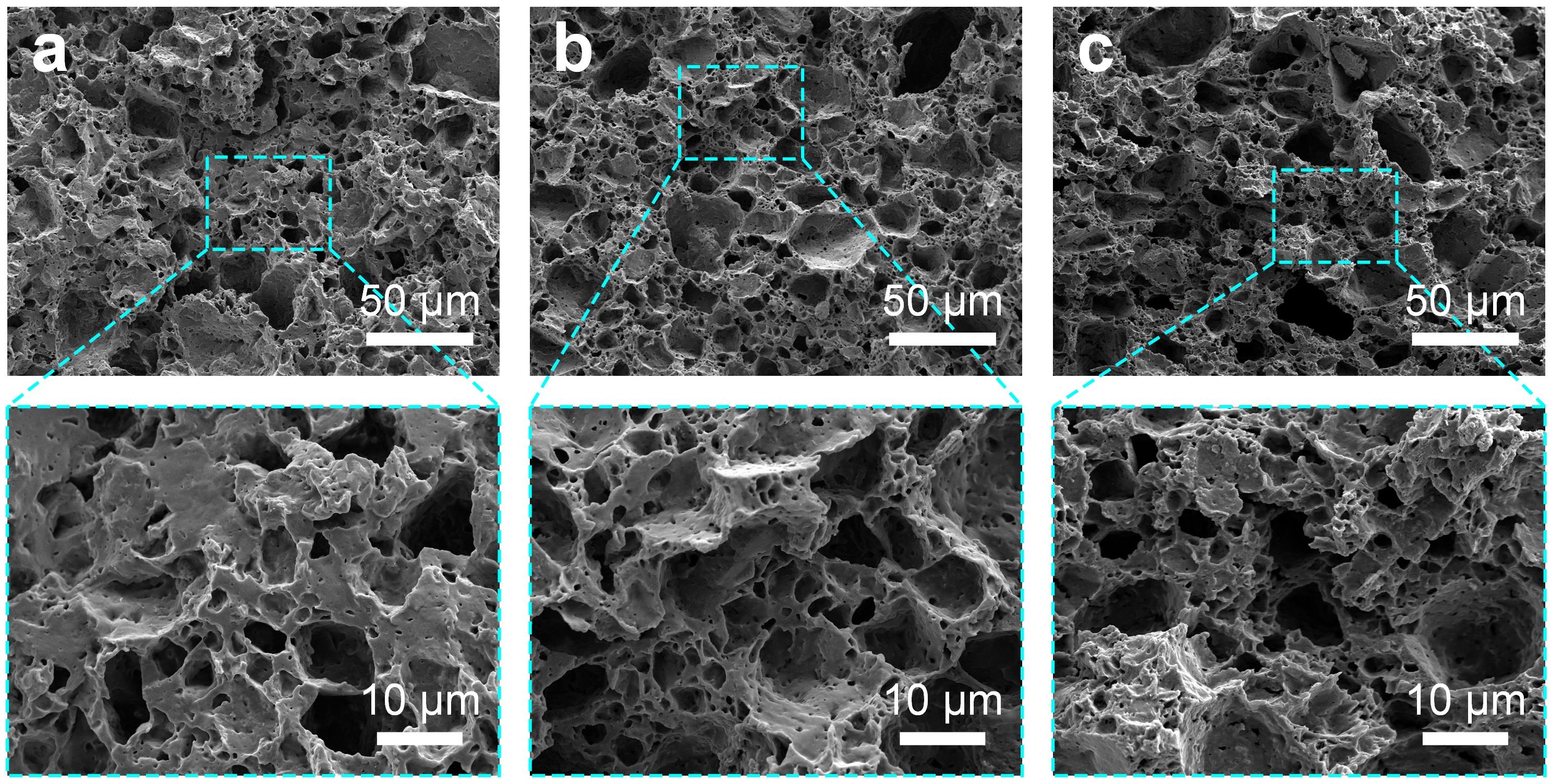


Fig. S9 Micromorphology of **a** TC-0, **b** TC-0.5, and **c** TC-1


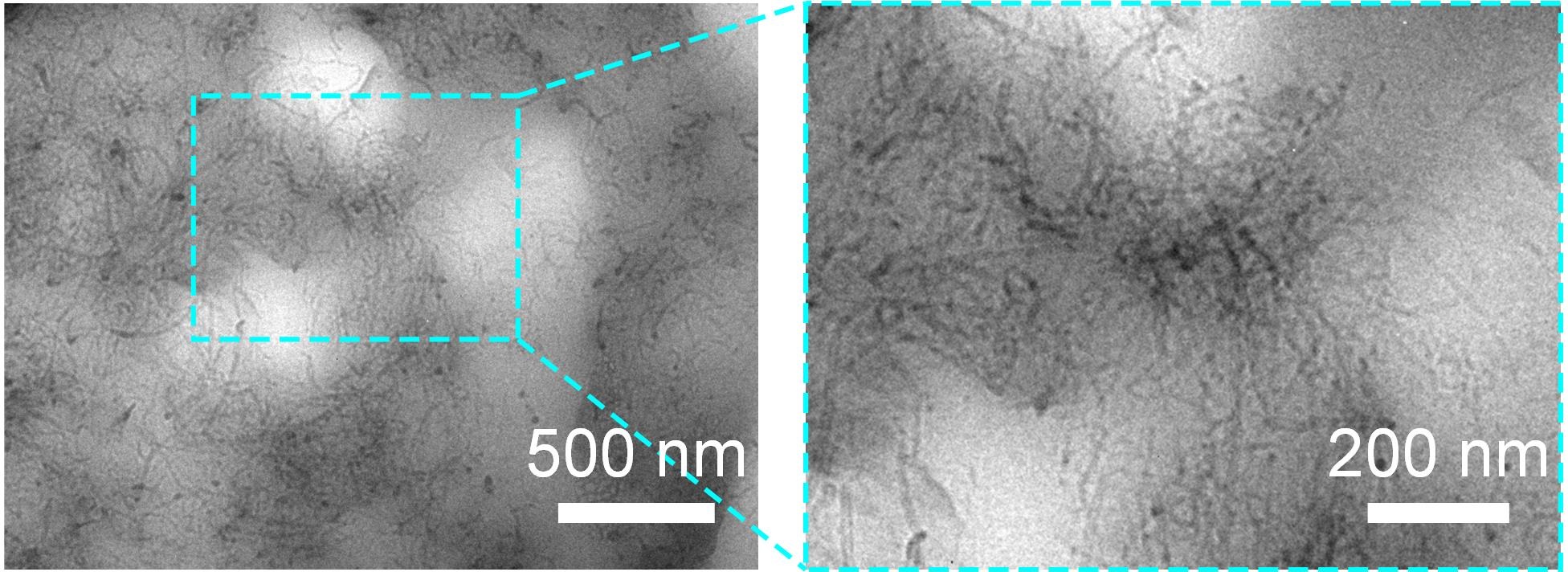


Fig. S10 Transmission electron microscopy (TEM) images of TC-1


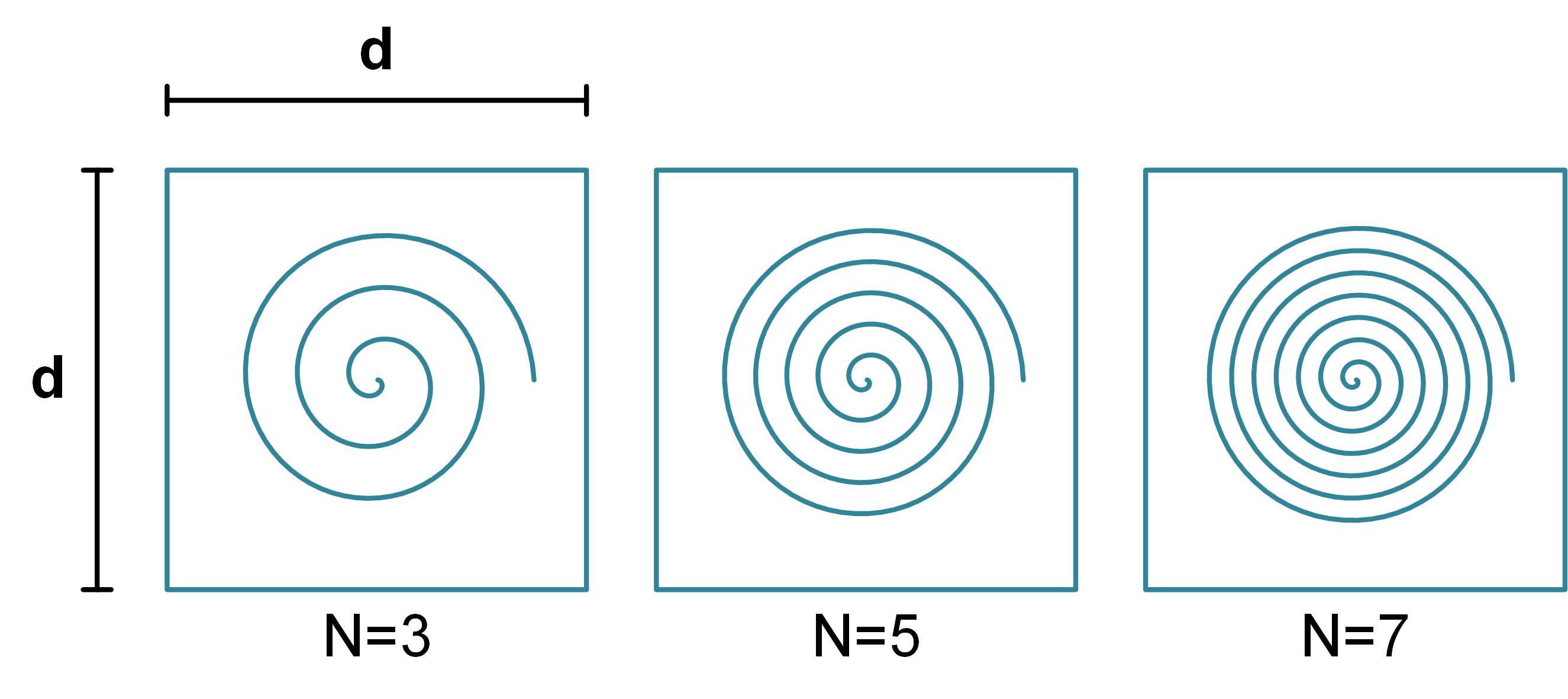


Fig. S11 The Archimedes spiral molds feature numbers of turns ($N$) of 3, 5, and 7. Each mold is square with a side length $d$ of 4 cm

In this work, inspired by the scorpion’s slit sensillum, three continuous arc-shaped structures comprising curved slits were designed to fabricate metal molds of Archimedean spirals with various numbers of turns ($N$ = 3, 5, and 7). The governing equation for these designs is expressed as:

$$\begin{aligned} r=a+b\theta\#\left( S1 \right) \end{aligned}$$

where $r$ represents the distance from the origin to a point on the curve, $\theta$ denotes the polar angle at that point, and $a$ and $b$ are constants. By setting the initial radius $a$ to zero, each spiral originates from the origin, simplifying the equation to:

$$\begin{aligned} r=b\theta\#\left( S2 \right) \end{aligned}$$

Given that each additional turn in an Archimedean spiral increases the radius by a constant value of $2\pi b$, the total angles for 3, 5, and 7 turns are calculated to be $6\pi$, $10\pi$, and $14\pi$, respectively. To ensure that the spiral fits within a circle with a diameter of 3 cm (radius of 1.5 cm) for the specified number of turns ($N$), the value of $b$ was determined as follows:

For $N=3$, the spiral equation yields:

$$\begin{aligned} r=\frac{1.5}{6\pi}\theta=0.0796\theta\#\left( S3 \right) \end{aligned}$$

For $N=5$, the spiral equation yields:

$$\begin{aligned} r=\frac{1.5}{10\pi}\theta=0.0477\theta\#\left( S4 \right) \end{aligned}$$

For $N=7$, the spiral equation yields:

$$\begin{aligned} r=\frac{1.5}{14\pi}\theta=0.0341\theta\#\left( S5 \right) \end{aligned}$$

The Archimedean spiral metal molds were used to prepare crack arrays on TCGS surfaces during the micro-extrusion compression molding process.


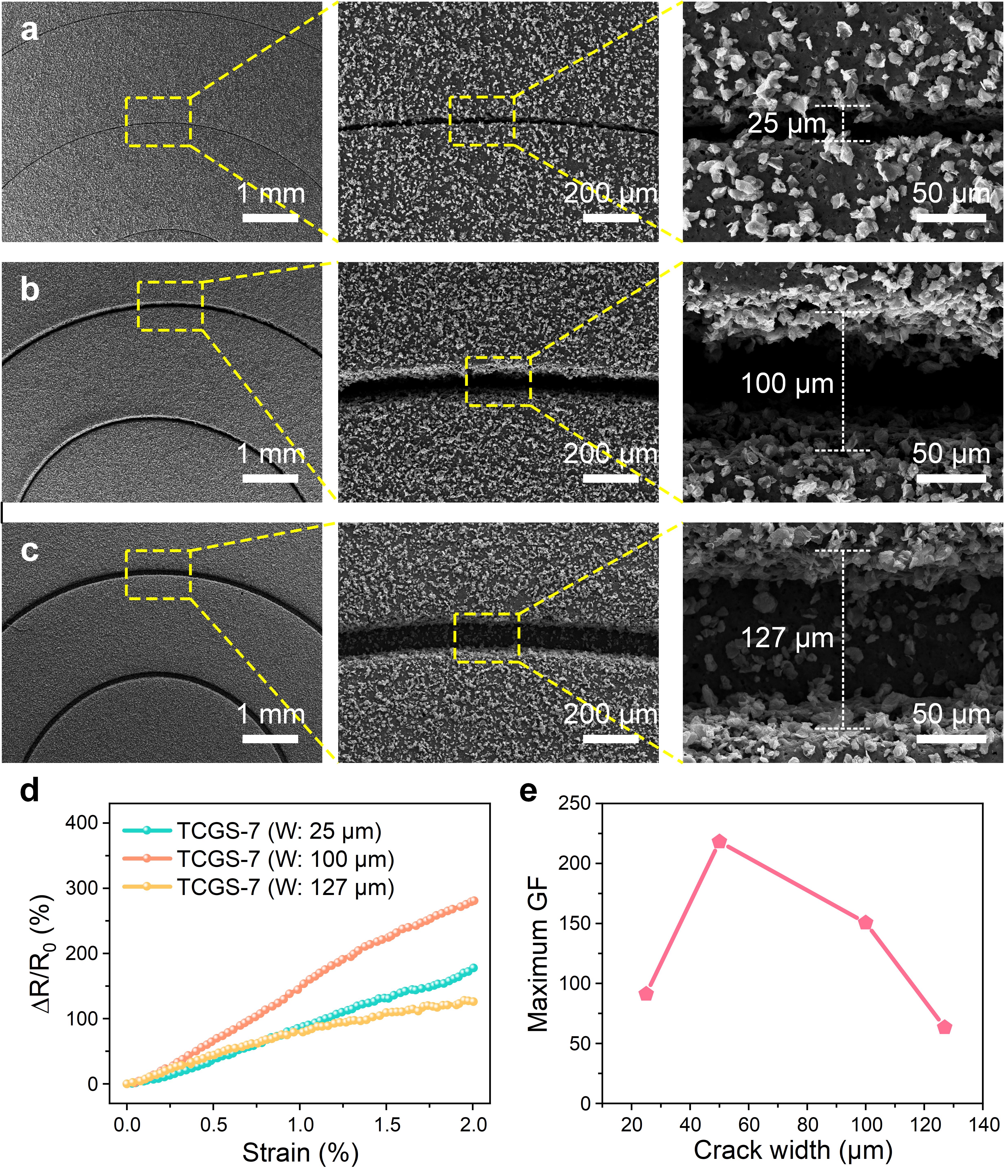


Fig. S12 SEM images and sensitivity analysis based on crack width for TCGS-7. SEM images showing crack widths of **a** 25 μm, **b** 100 μm, and **c** 127 μm. **d** Sensitivity response to strain for crack widths of 25 μm, 50 μm, 100 μm and 127 μm in TCGS-7. **e** The maximum GF versus crack width


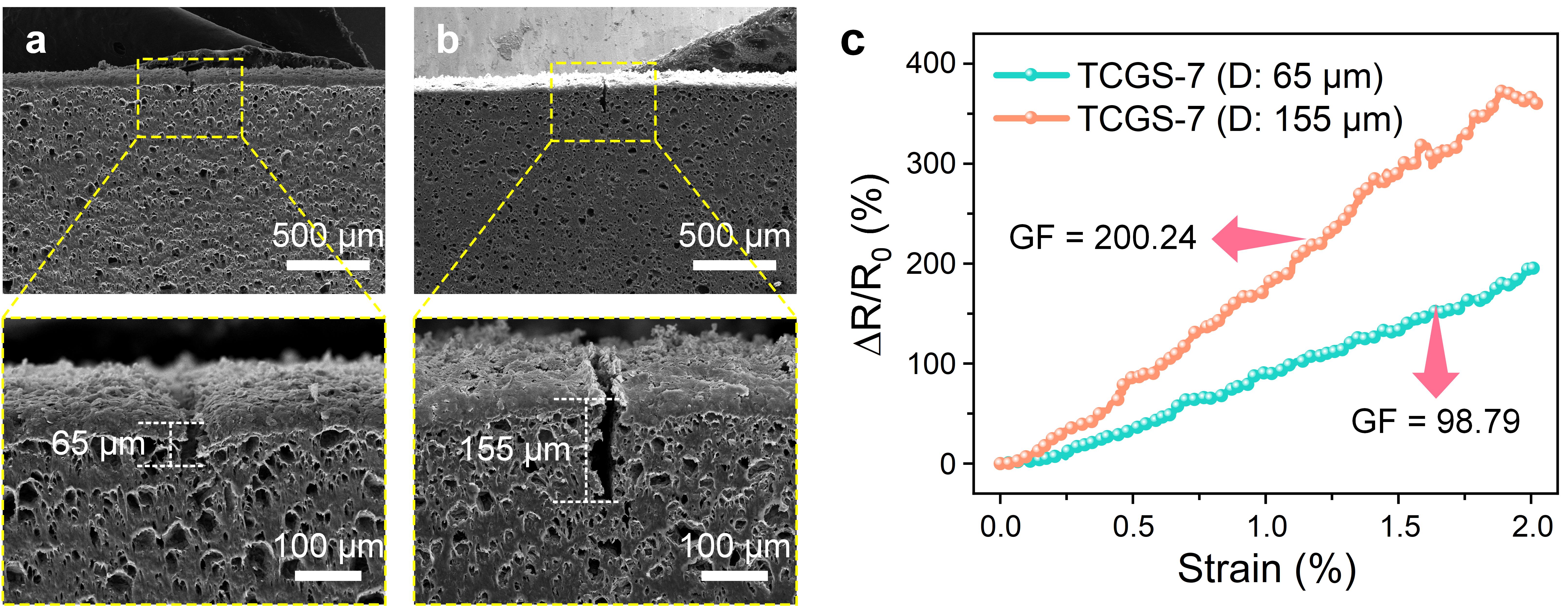


Fig. S13 SEM images and sensitivity analysis based on crack depth for TCGS-7. SEM images showing crack depths of **a** 65 μm and **b**155 μm. **c** Sensitivity response to strain for crack depths of 65 μm and 155 μm in TCGS-7


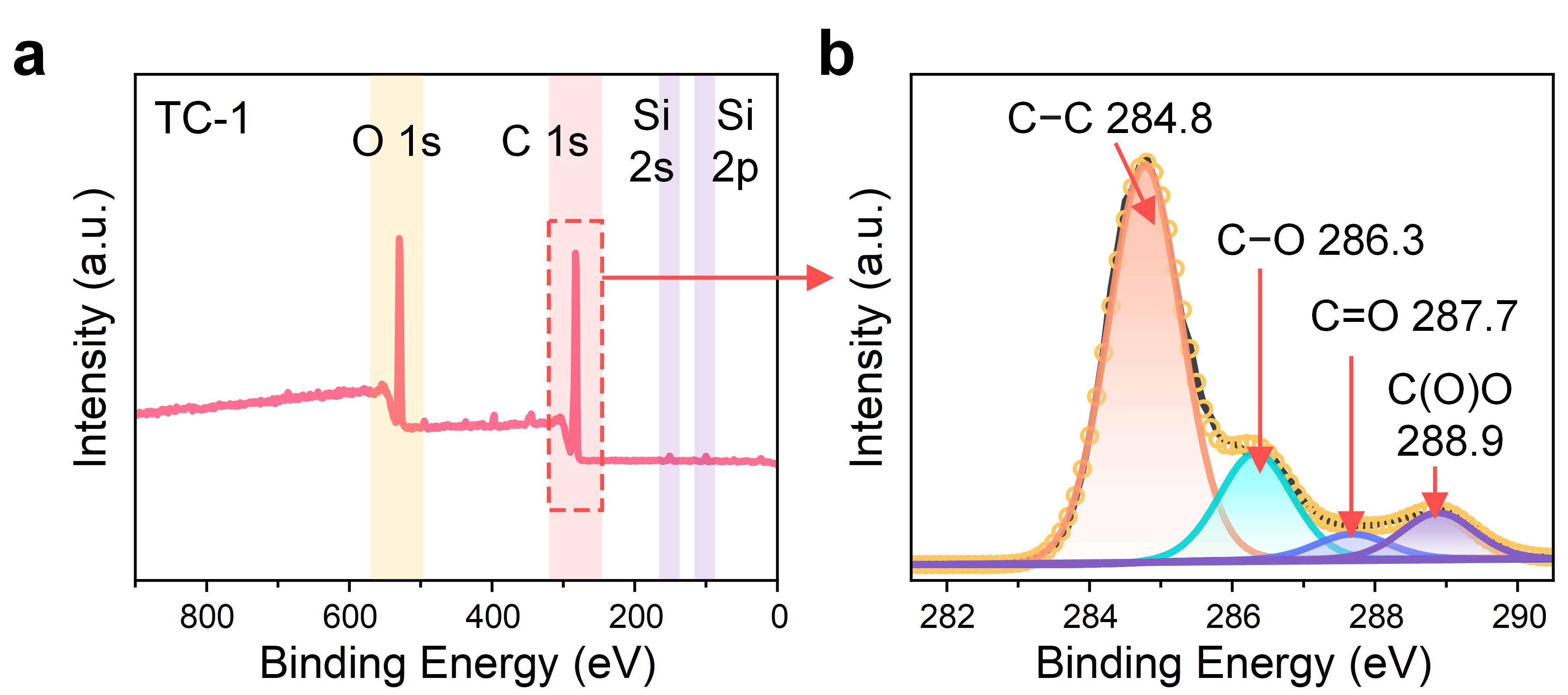


Fig. S14 XPS spectra. **a** Survey spectrum of TC-1. **b** C 1s XPS spectrum of TC-1


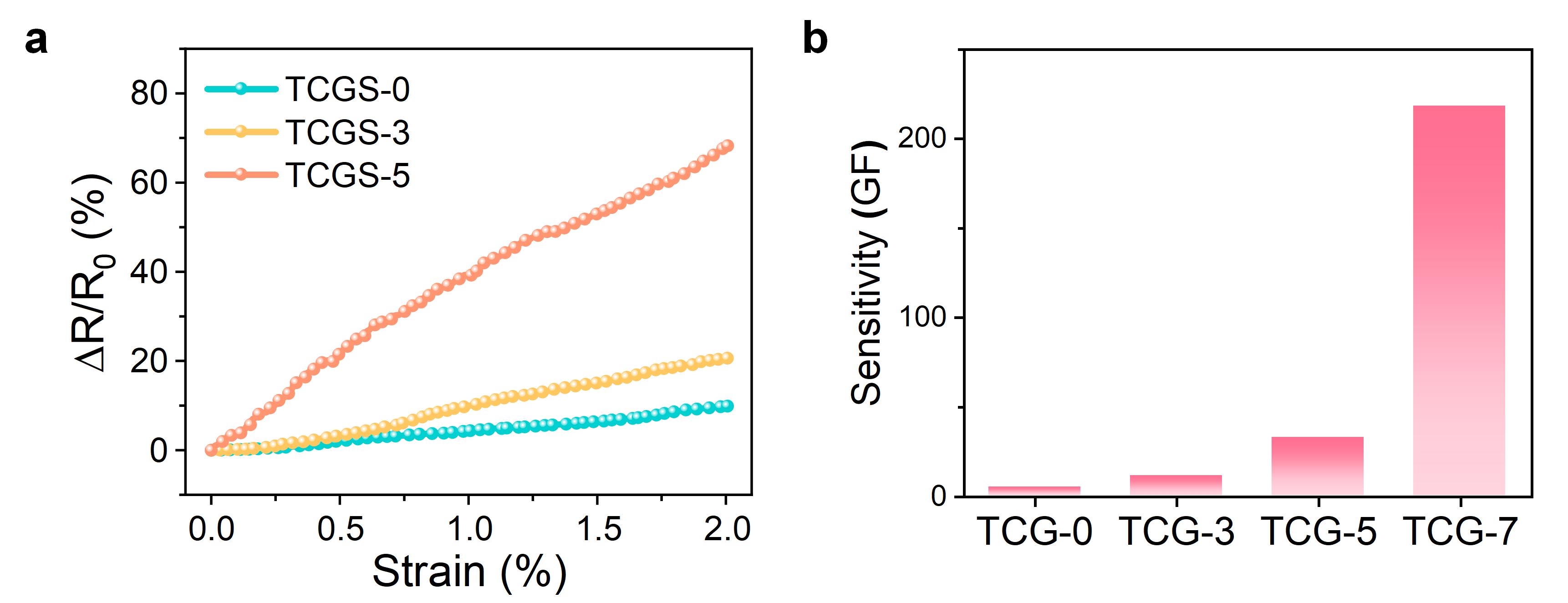


Fig. S15 The strain sensing performance. **a** The resistance changes for TCGS-0, TCGS-3, and TCGS-5 within the strain range of 0% to 2%. **b** Comparison of maximum gauge factor (GF) among TCGS-0, TCGS-3, TCGS-5, and TCGS-7
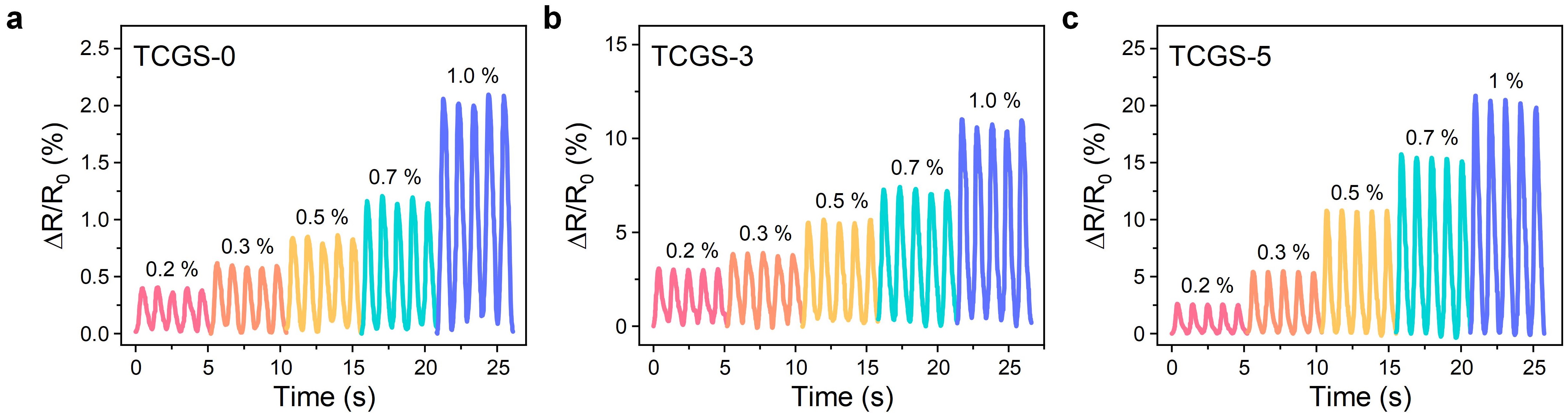


Fig. S16 The ΔR/R_0_-strain curves of **a** TCGS-0, **b** TCGS-3, and **c** TCGS-5 at strains of 0.2%, 0.3%, 0.5%, 0.7%, and 1%


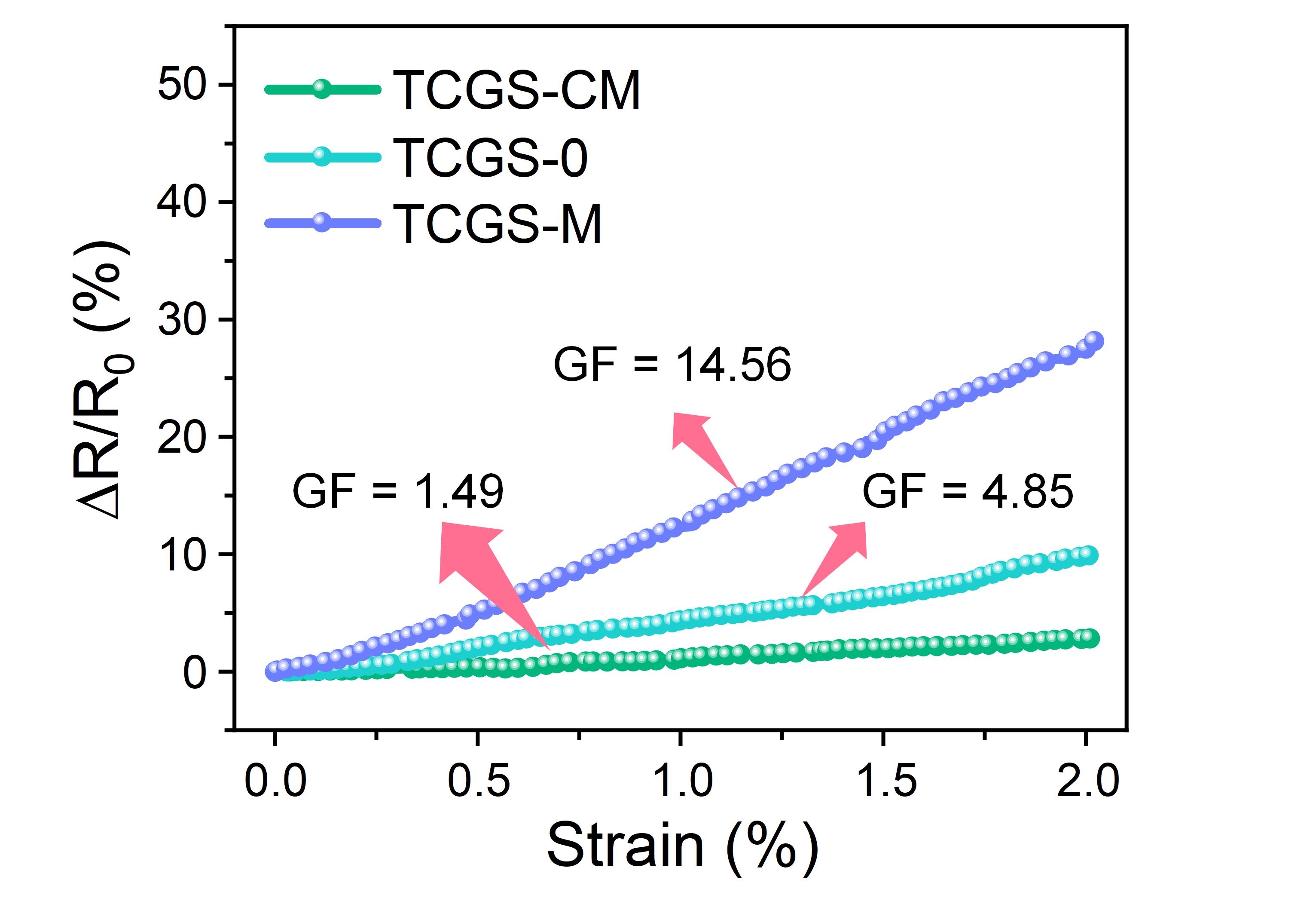


Fig. S17 The strain sensing comparison: TCGS-CM (without cracks or micropores), TCGS-0 (with micropores but without cracks), and TCGS-M (with cracks but without micropores)


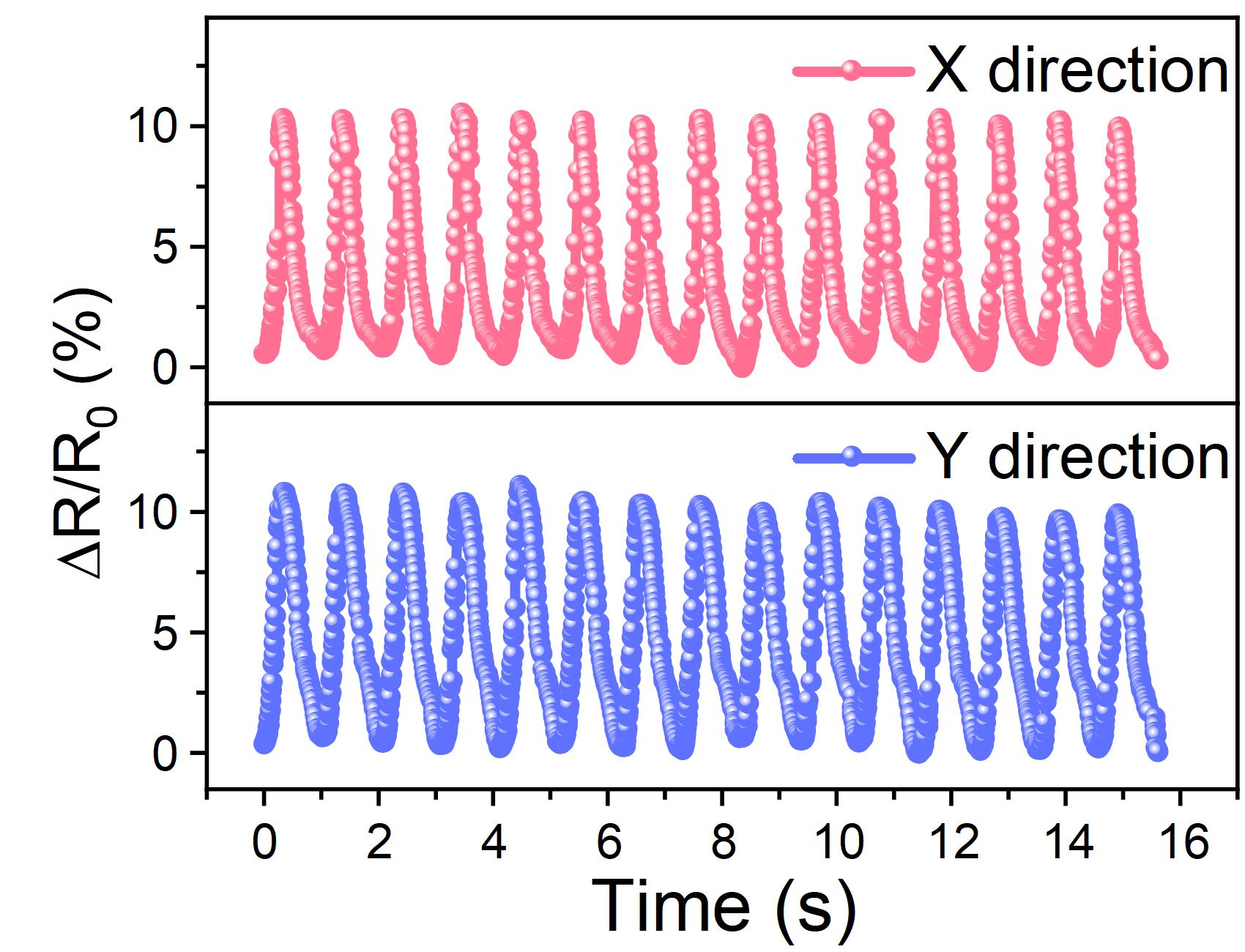


Fig. S18 Electrical response signals of TCGS-7 to forces applied in the X and Y directions. The stretching frequency is 1 Hz, with a strain of 0.3%. The output signals showing the resistance changes are almost the same in both directions


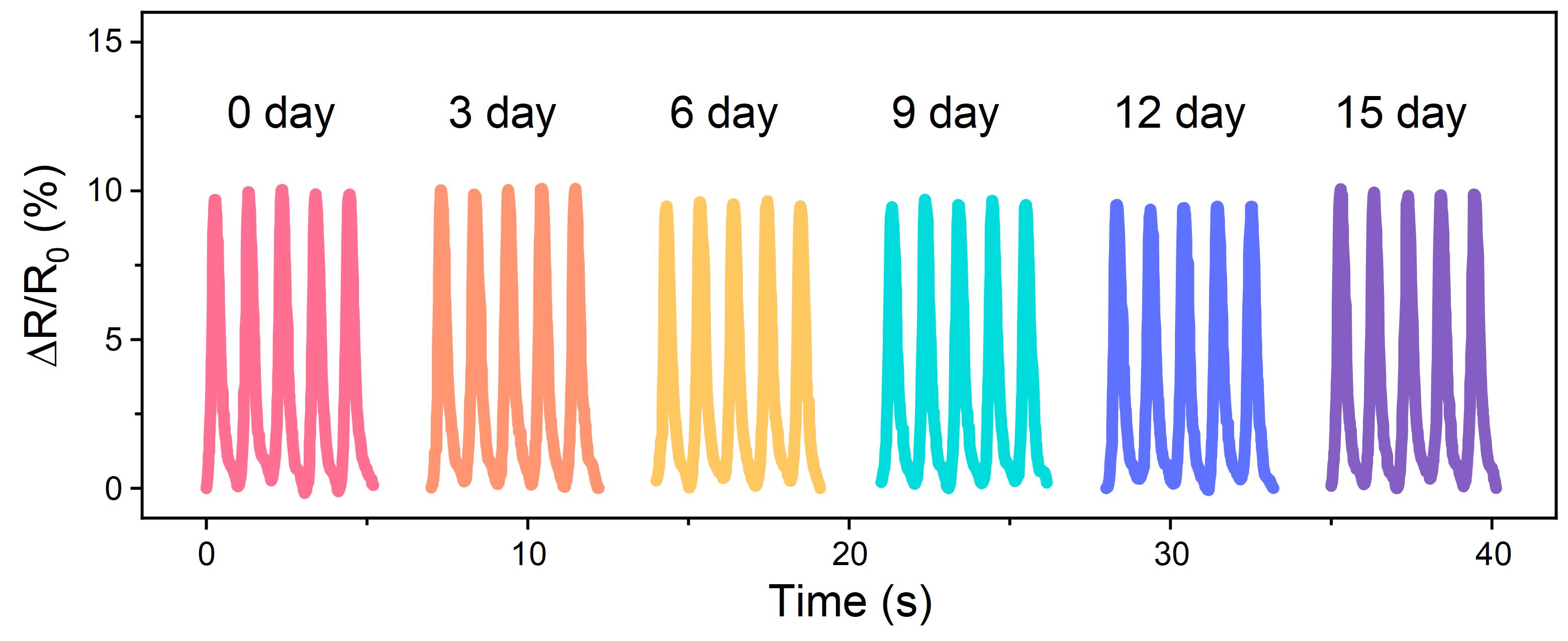


Fig. S19 Strain sensing stability of TCGS-7 immersed in water for different days


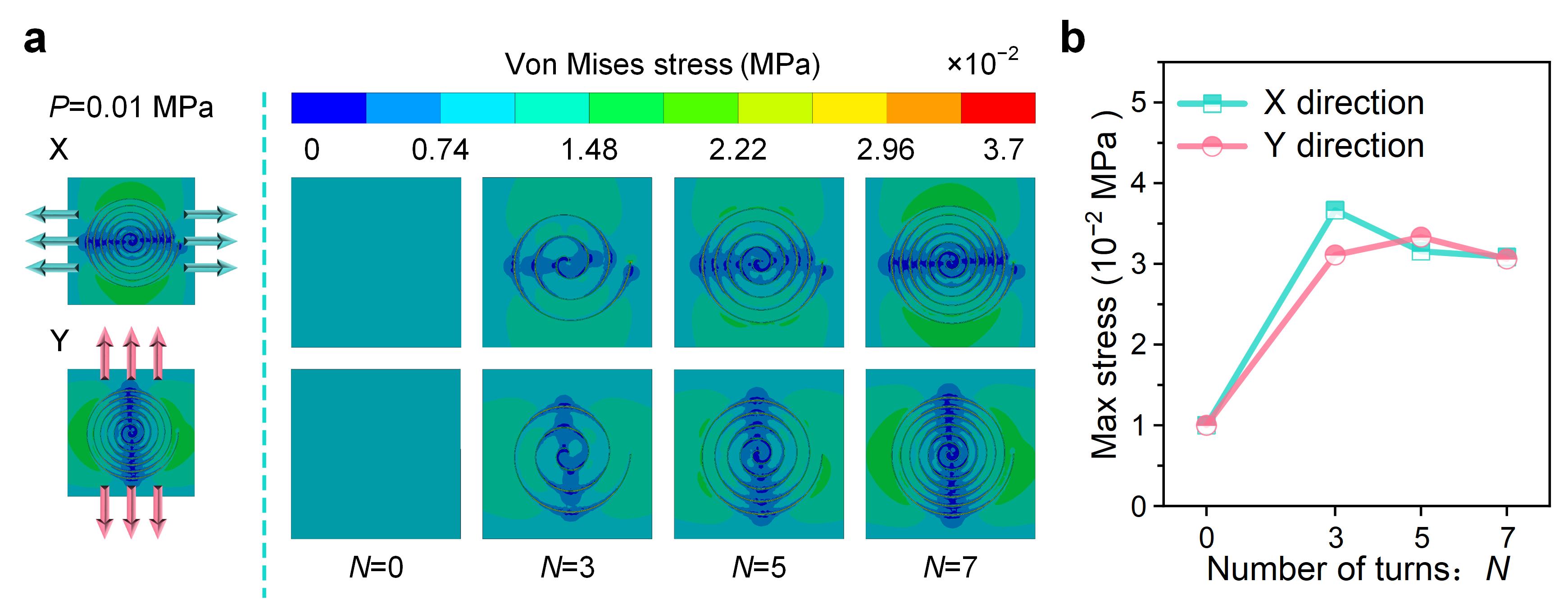


Fig. S20 FEA simulations of models featuring Archimedean spiral cracks with varying numbers of turns ($N=0, 3, 5, and 7$). **a** Von Mises stress distribution within the models, with pressures of 0.01 MPa applied at both ends independently along the X and Y directions. **b** The correlation between $N$ and the maximum stress along the X and Y directions


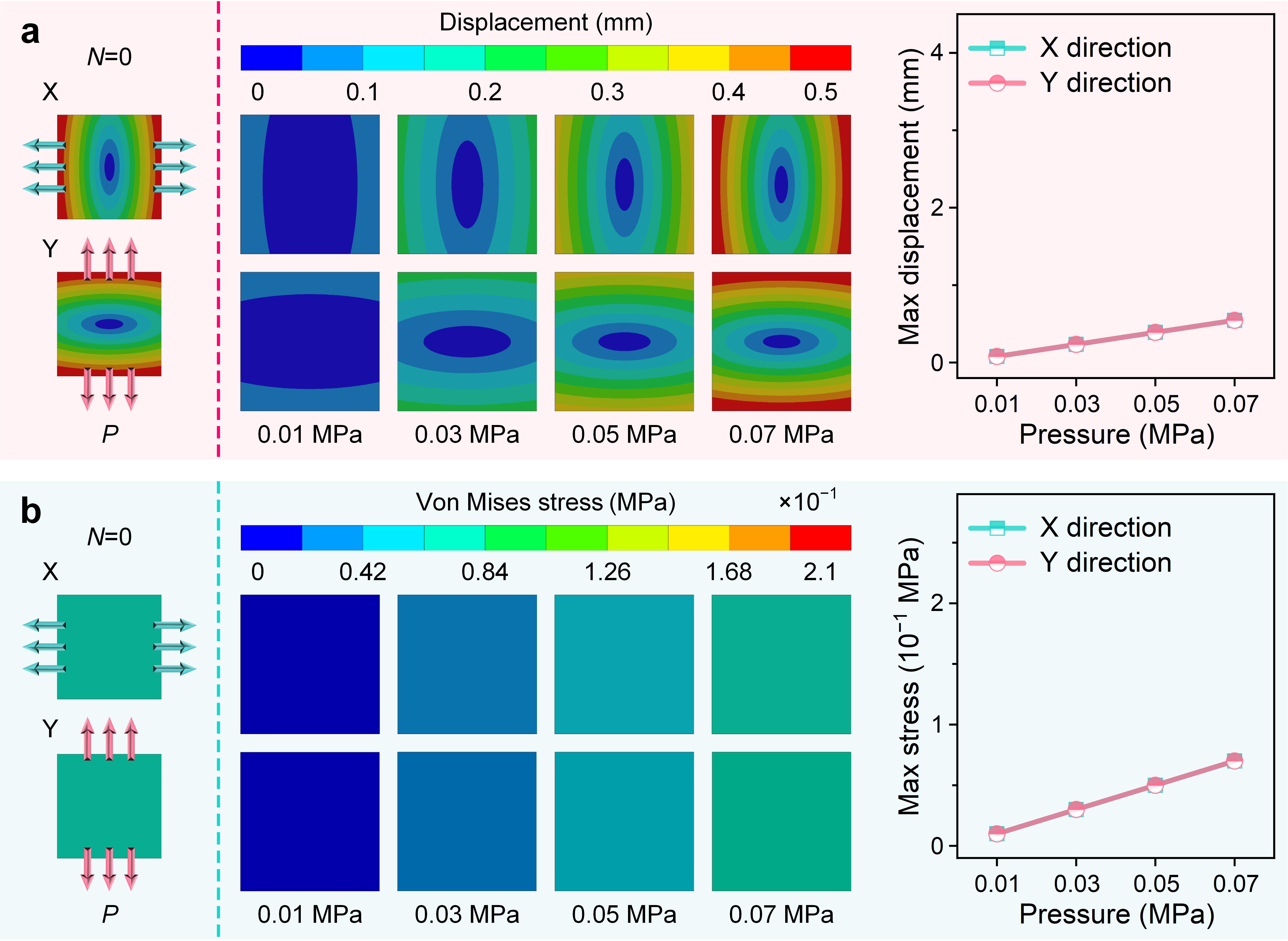


Fig. S21 FEA simulation for TCGS-0 ($N=0$). **a** Displacement variations under different pressures applied in the X and Y direction (0.01 MPa, 0.03 MPa, 0.05 MPa, and 0.07 MPa), and the relationship between maximum displacement and pressure. **b** Von Mises stress distribution under different pressures applied separately in the X and Y directions (0.01 MPa, 0.03 MPa, 0.05 MPa, and 0.07 MPa), and the relationship between maximum stress and pressure


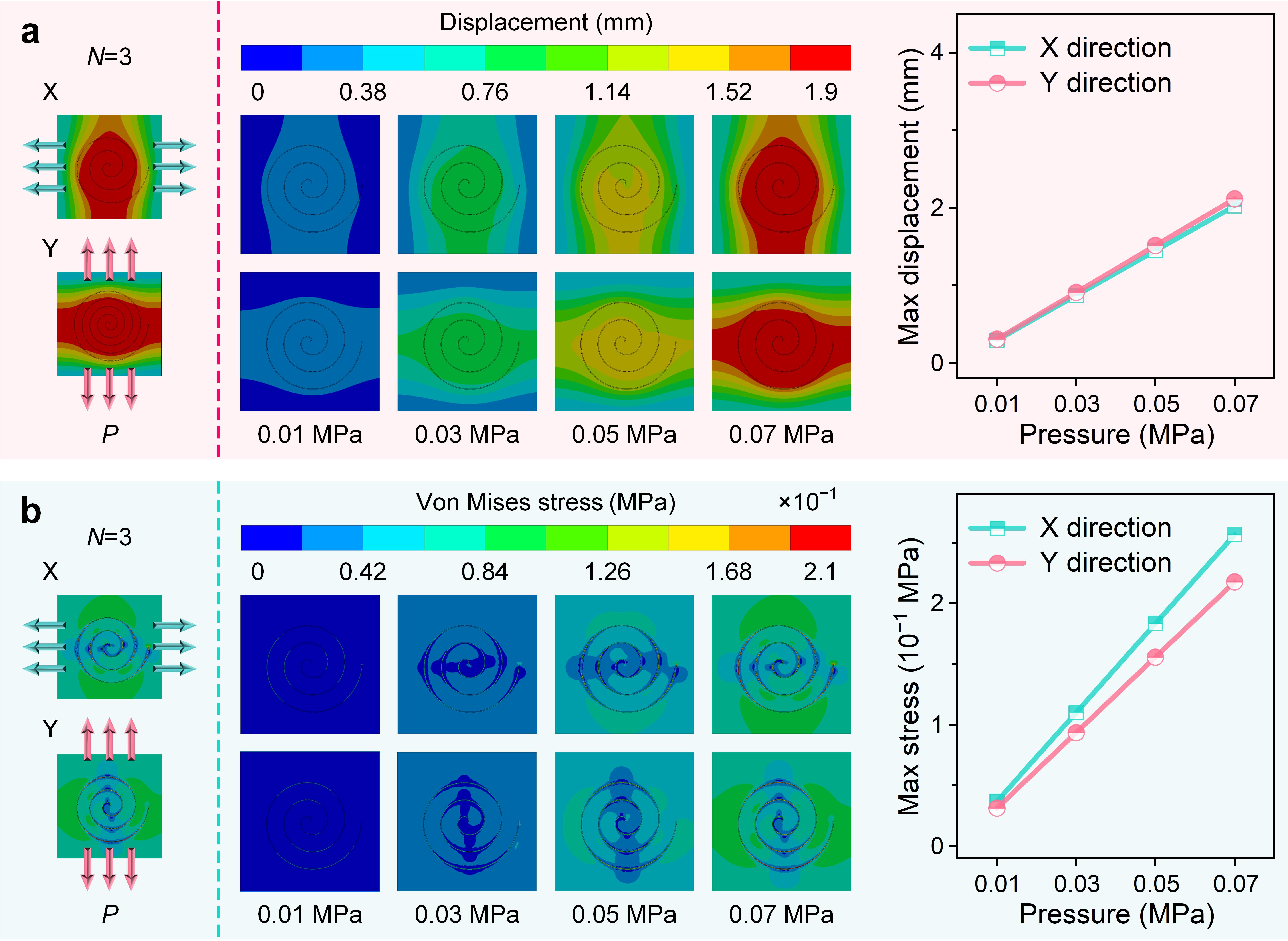


Fig. S22 FEA simulation for TCGS-3 ($N=3$). **a** Displacement variations under different pressures applied in the X and Y direction (0.01 MPa, 0.03 MPa, 0.05 MPa, and 0.07 MPa), and the relationship between maximum displacement and pressure. **b** Von Mises stress distribution under different pressures applied separately in the X and Y directions (0.01 MPa, 0.03 MPa, 0.05 MPa, and 0.07 MPa), and the relationship between maximum stress and pressure


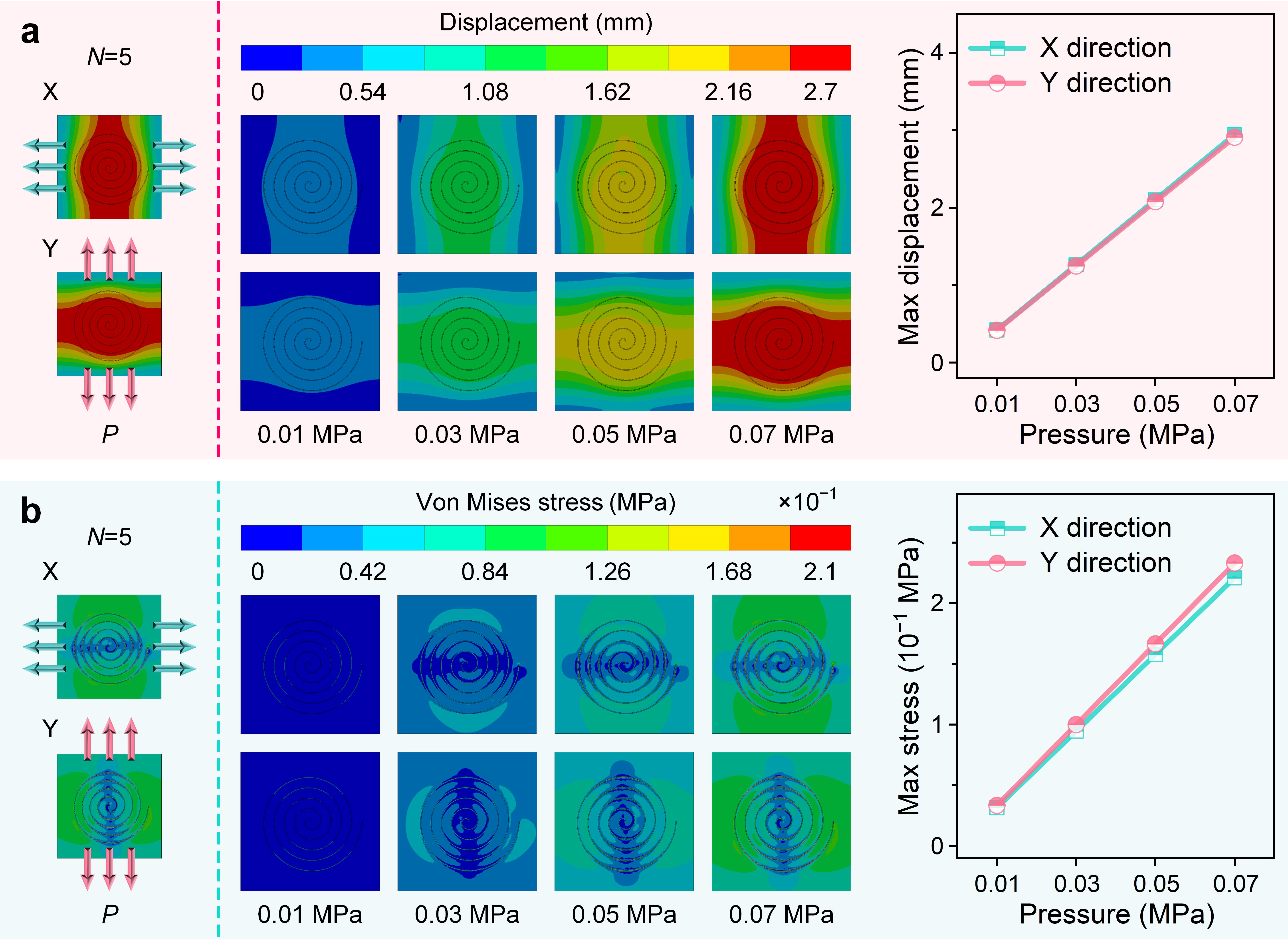


Fig. S23 FEA simulation for TCGS-5 ($N=5$). **a** Displacement variations under different pressures applied in the X and Y direction (0.01 MPa, 0.03 MPa, 0.05 MPa, and 0.07 MPa), and the relationship between maximum displacement and pressure. **b** Von Mises stress distribution under different pressures applied separately in the X and Y directions (0.01 MPa, 0.03 MPa, 0.05 MPa, and 0.07 MPa), and the relationship between maximum stress and pressure


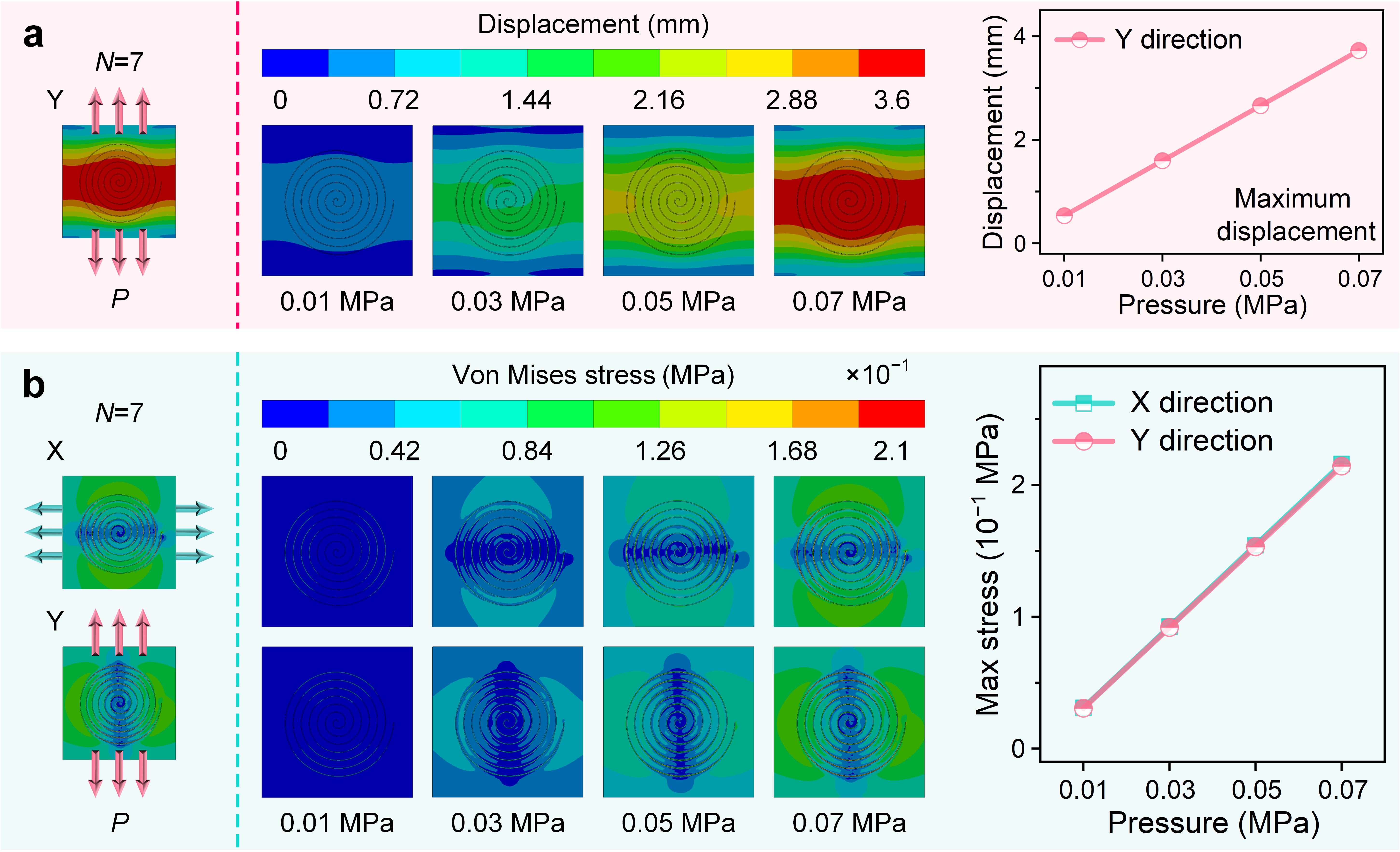


Fig. S24 FEA simulation for TCGS-7 ($N=7$). **a** Displacement variations under different pressures applied in the Y direction (0.01 MPa, 0.03 MPa, 0.05 MPa, and 0.07 MPa), and the relationship between maximum displacement and pressure. **b** Von Mises stress distribution under different pressures applied separately in the X and Y directions (0.01 MPa, 0.03 MPa, 0.05 MPa, and 0.07 MPa), and the relationship between maximum stress and pressure


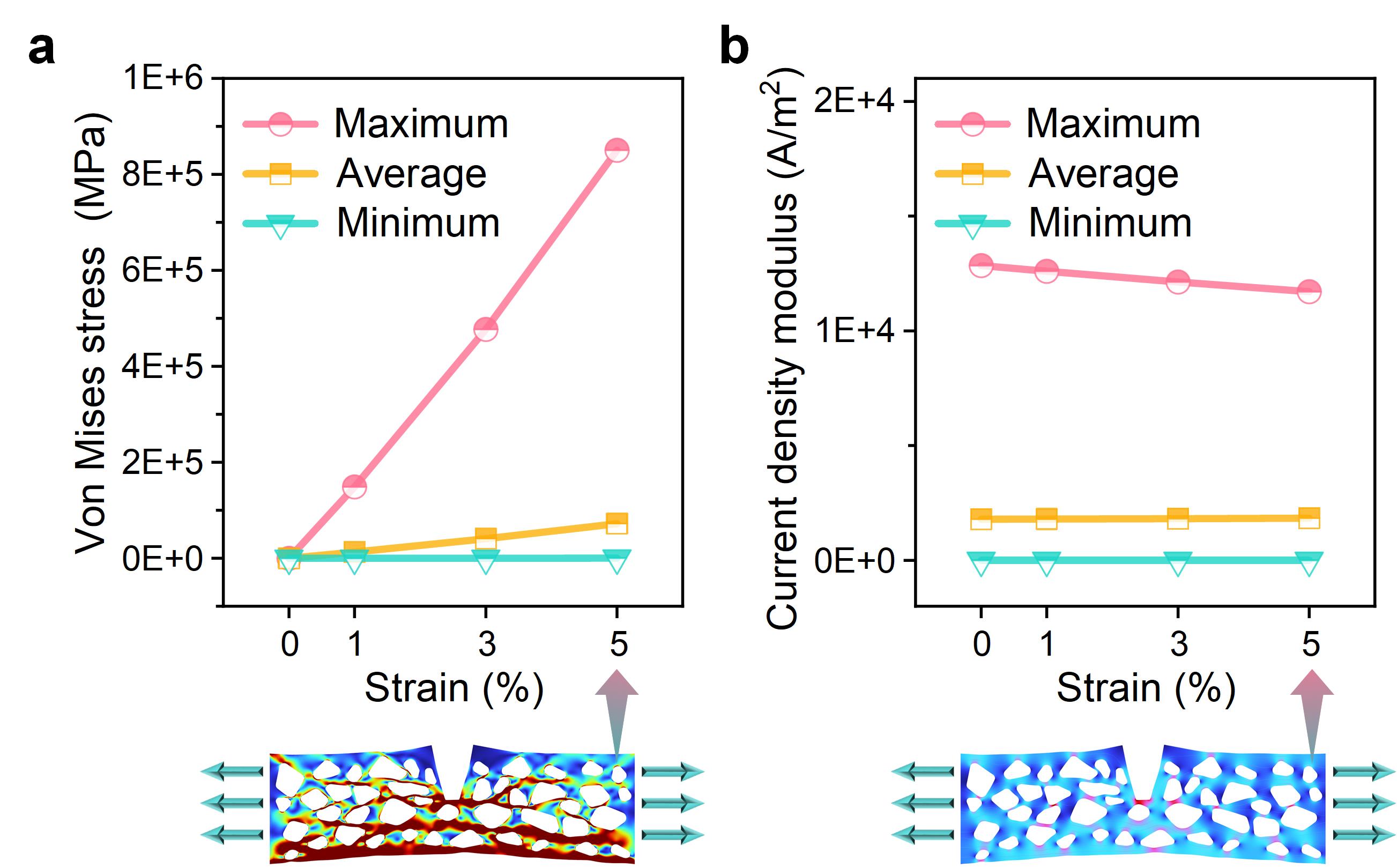


Fig. S25 FEA simulation for micropore-crack synergistic structure. **a** The relationship between Von Mises stress distribution and strain. **b** The relationship between current density modulus and strain


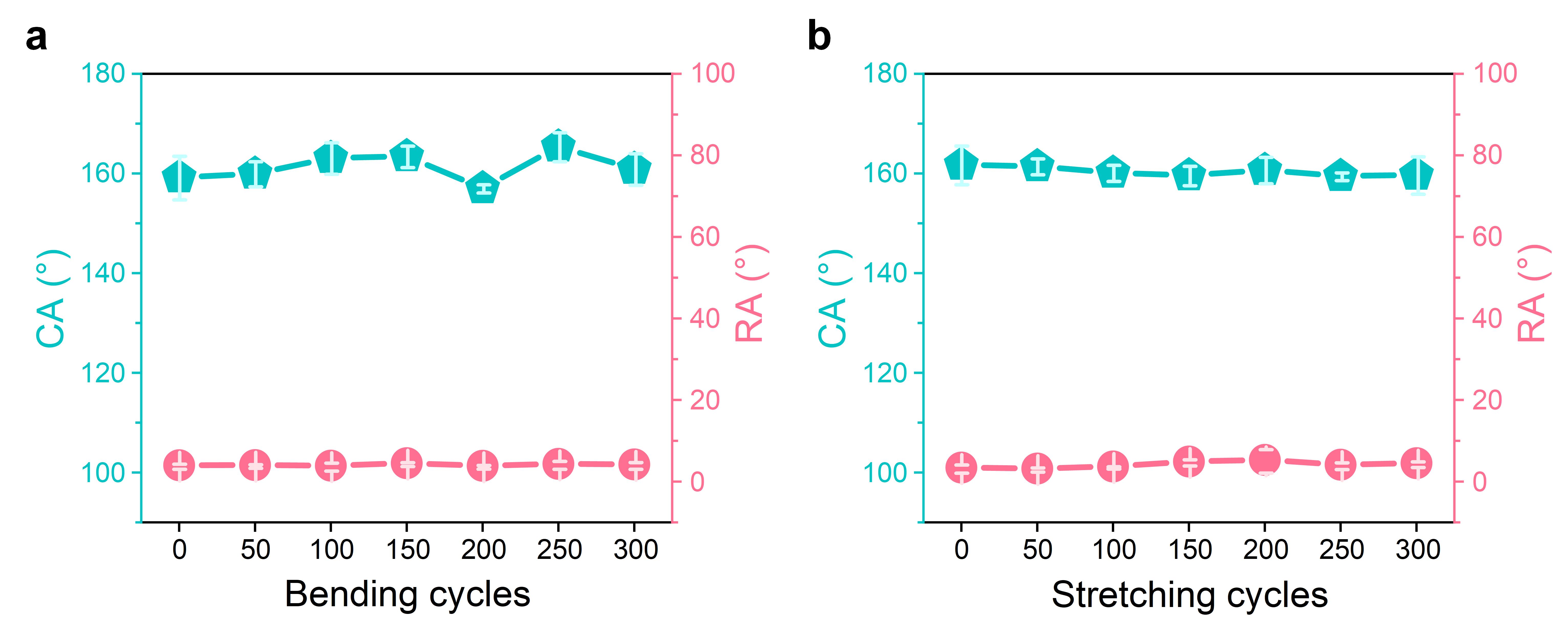


**Fig. S26** Durability of wettability of TCGS under mechanical deformation. **a** Bending test. **b** Stretching test


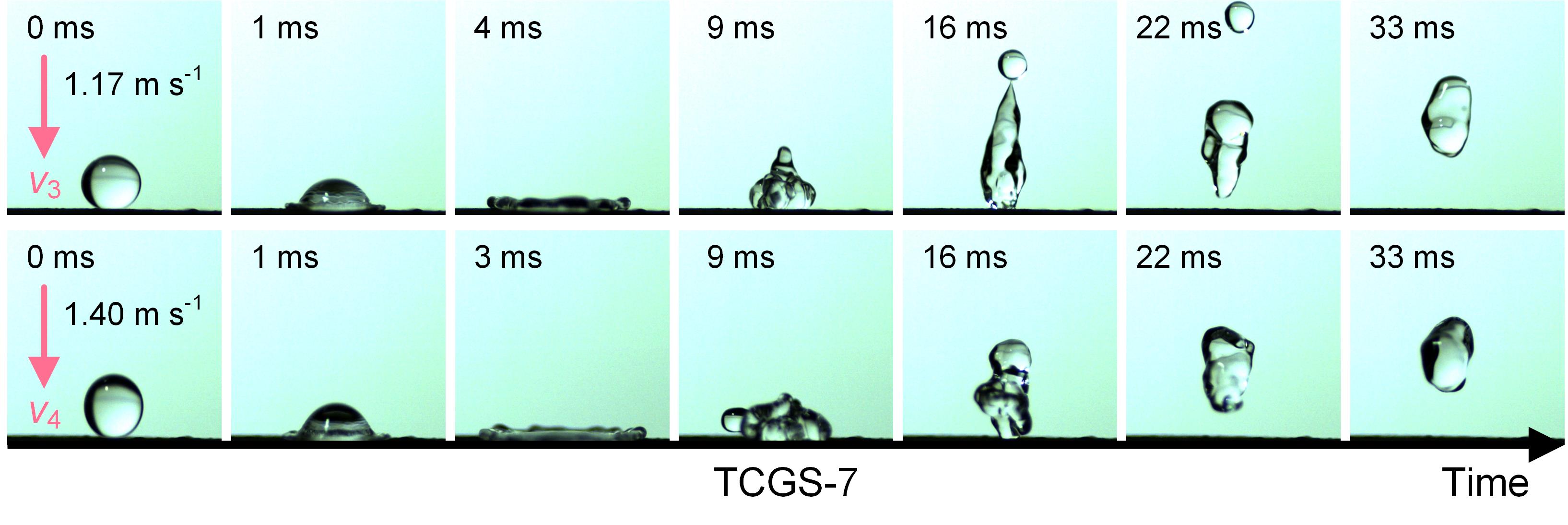


Fig. S27 Selected snapshots showing droplets impacting the TCGS-7 surface at velocities of 1.17 m s^−1^ (top) and 1.40 m s^−1^ (bottom)


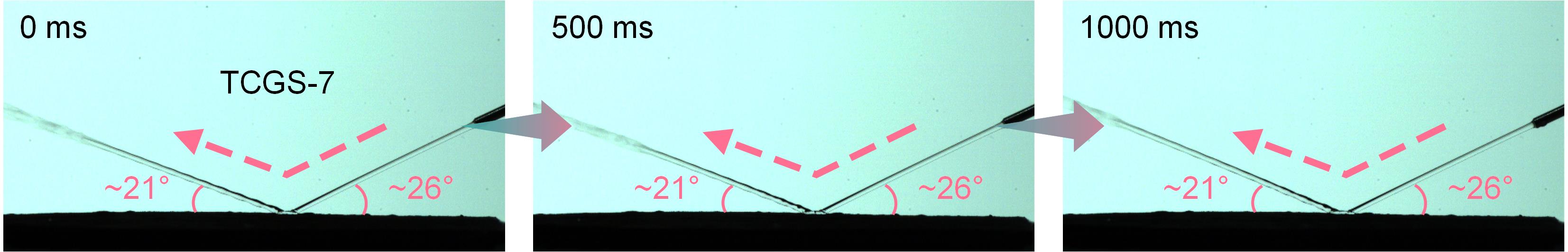


Fig. S28 Water jet impact test. The velocity of water jet is maintained at 7.5 m s^−1^ and the diameter of water jet is about 300 μm


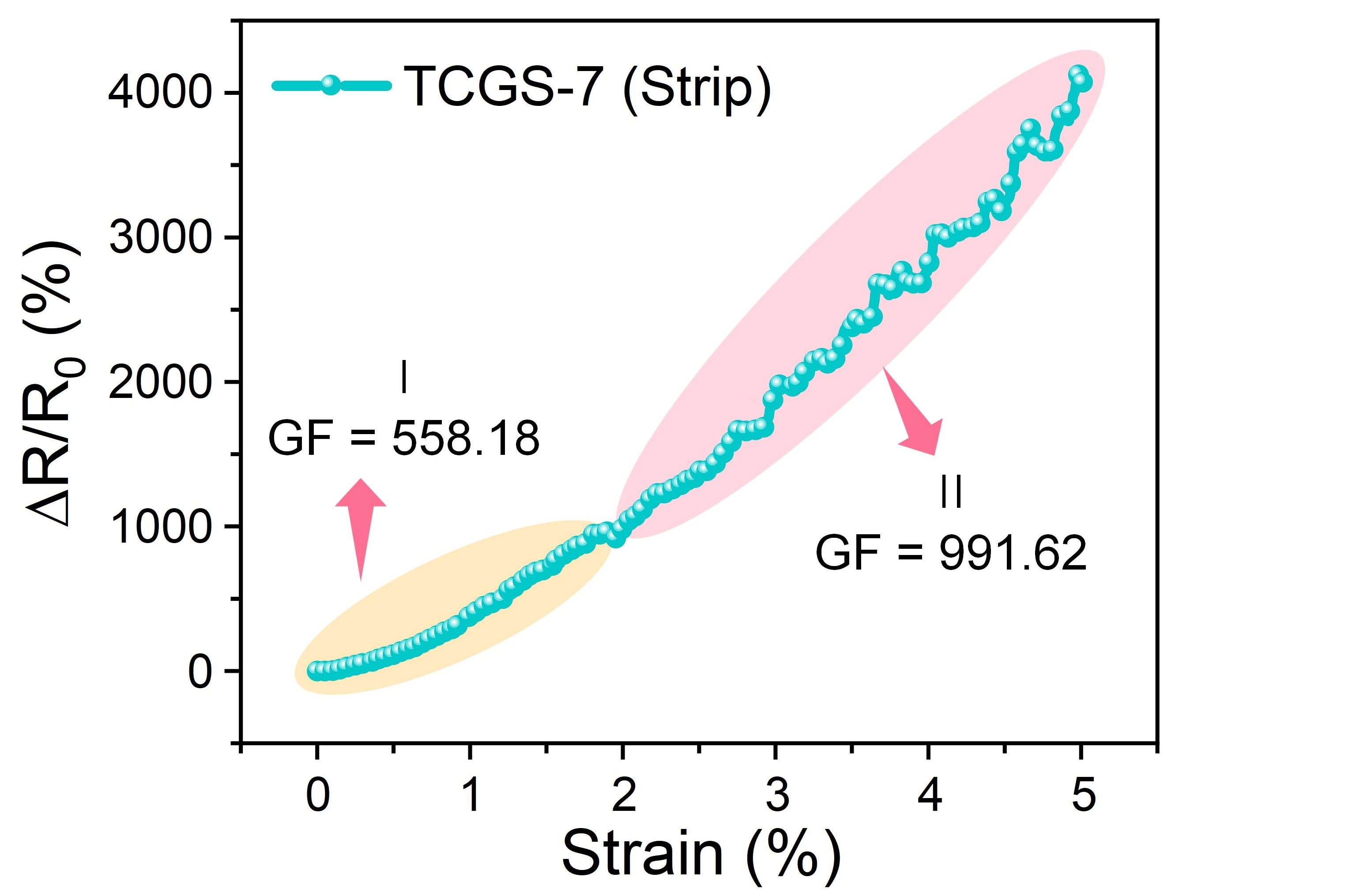


Fig. S29 Strain-sensitivity performance of the trimmed 0.5 cm × 4 cm TCGS-7 strip


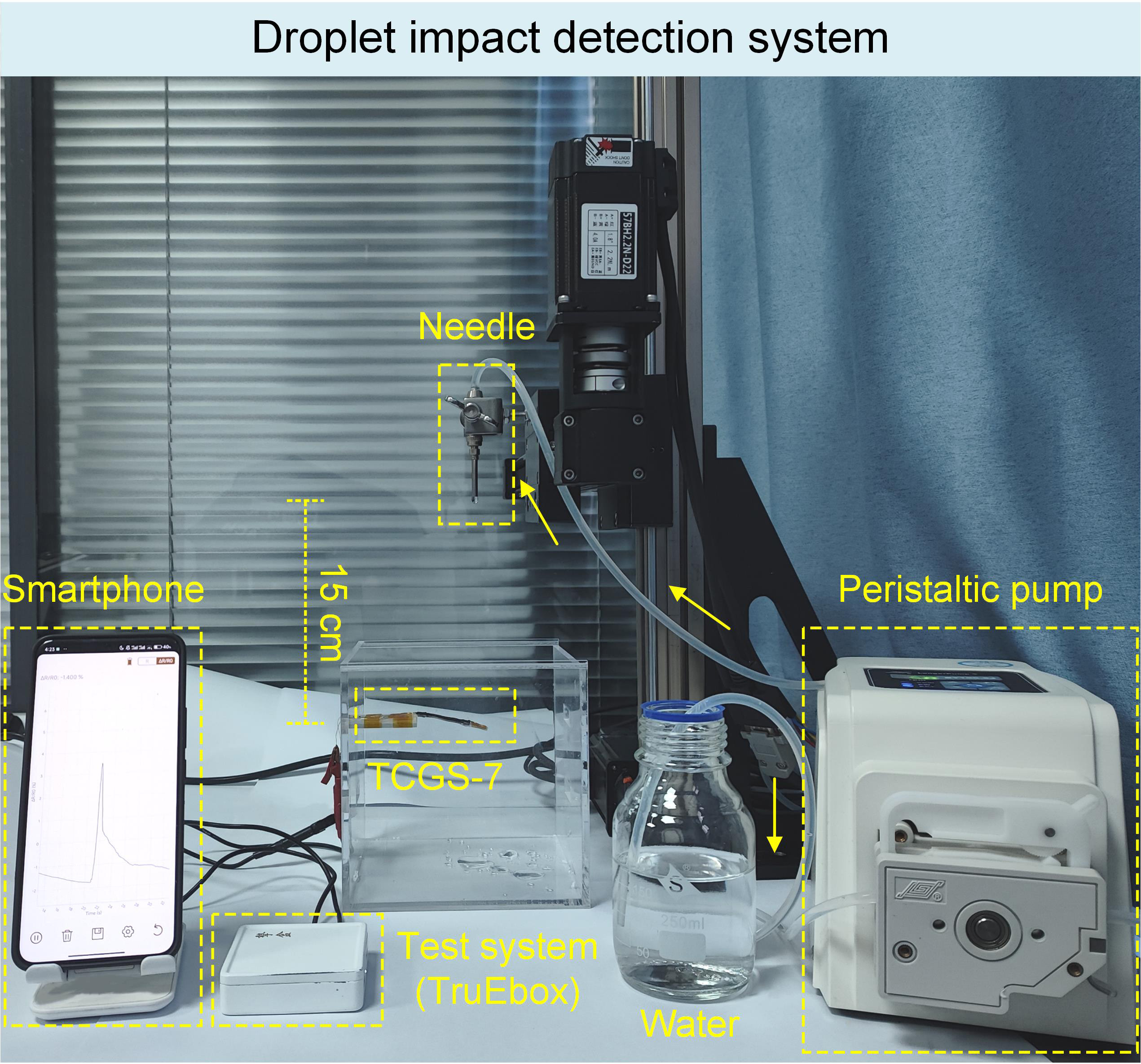


Fig. S30 Water droplet impact detection system

In the process, TCGS-7 is cut into a 0.5 cm × 4cm strip sensor, retaining part of the its inherent cracks and micropores, and is fixed on the bracket as a cantilever beam. The solution is transported from a container using a peristaltic pump (BT100-2J). As the droplet falls from the needle and impacts the TCGS-7 surface, it causes deformation in the cracks and micropores of the sensor, which in turn changes its resistance. The resistance testing system (TruEbox) captures this change in resistance and transmits the signal to a smartphone for analysis. By adjusting the diameter of the needle, the size of the droplets can be controlled. Additionally, altering the distance between the needle and the sensor allows for control over the fall height of the droplets, enabling the testing of signal variations under different impact intensities.


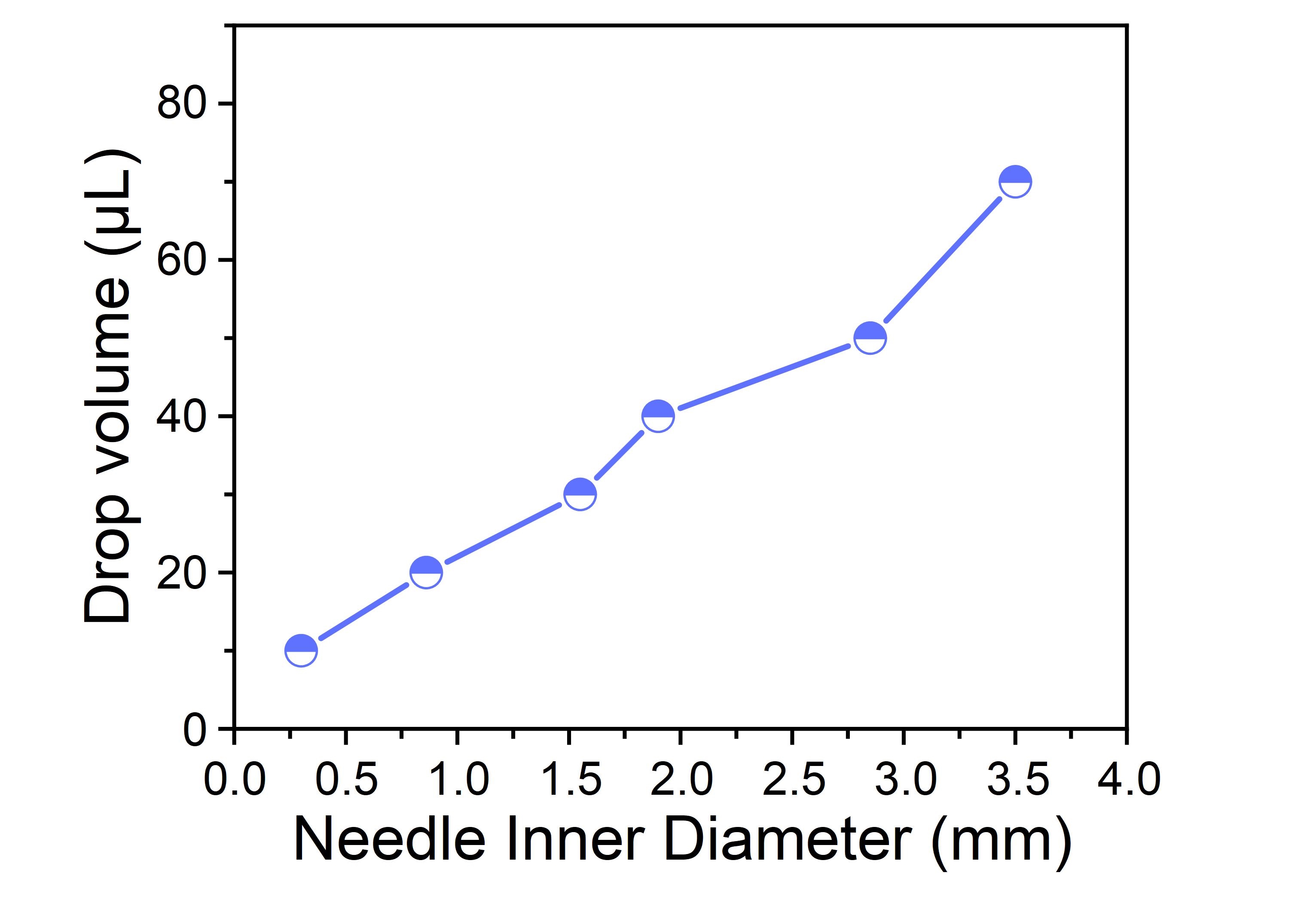


Fig. S31 Relationship between needle inner diameter and droplet volume


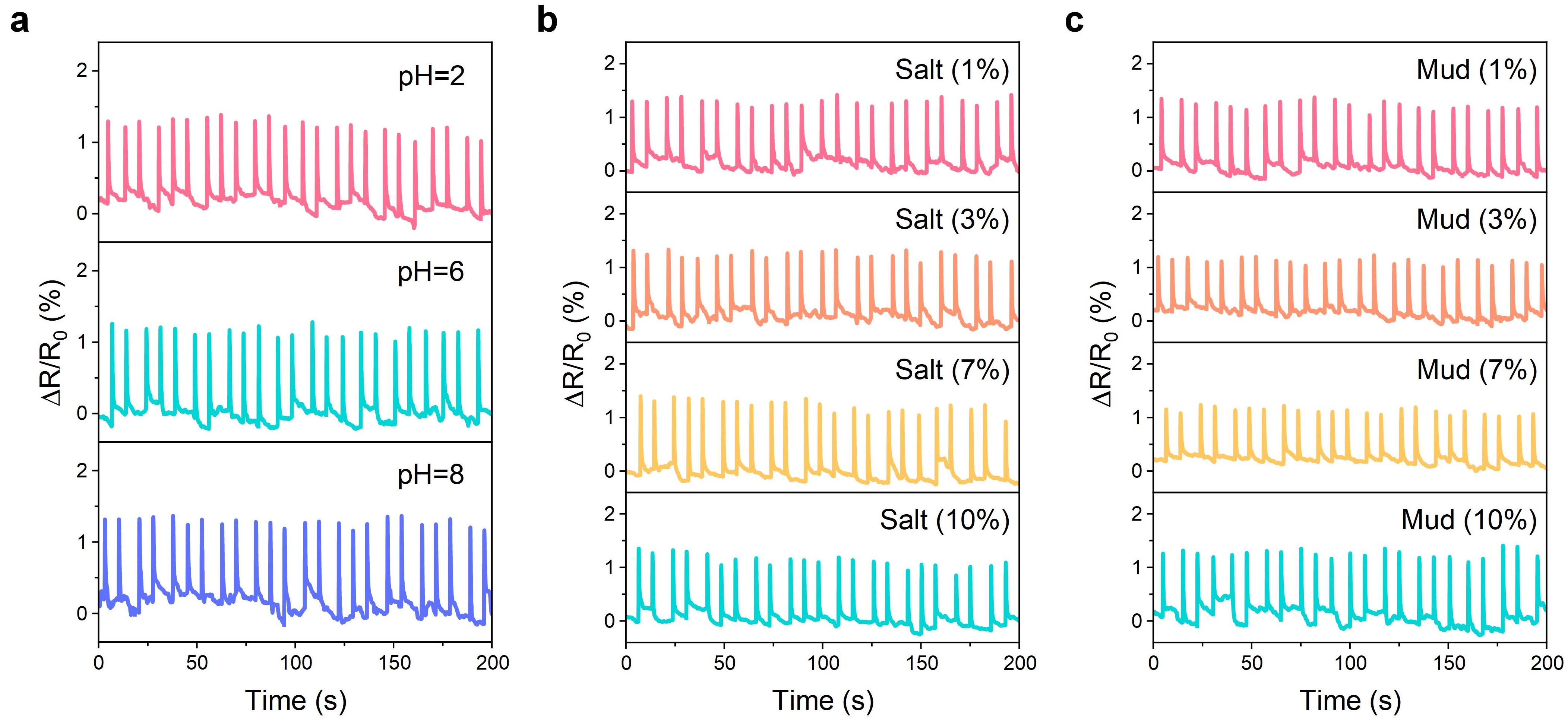


Fig. S32 Electrical response signal to various types of liquid droplets. **a** pH levels: 2, 6, 8. **b** Different concentrations of salt solution: 1%, 3%, 7%, and 10%. **c** Different concentrations of mud: 1%, 3%, 7%, and 10%


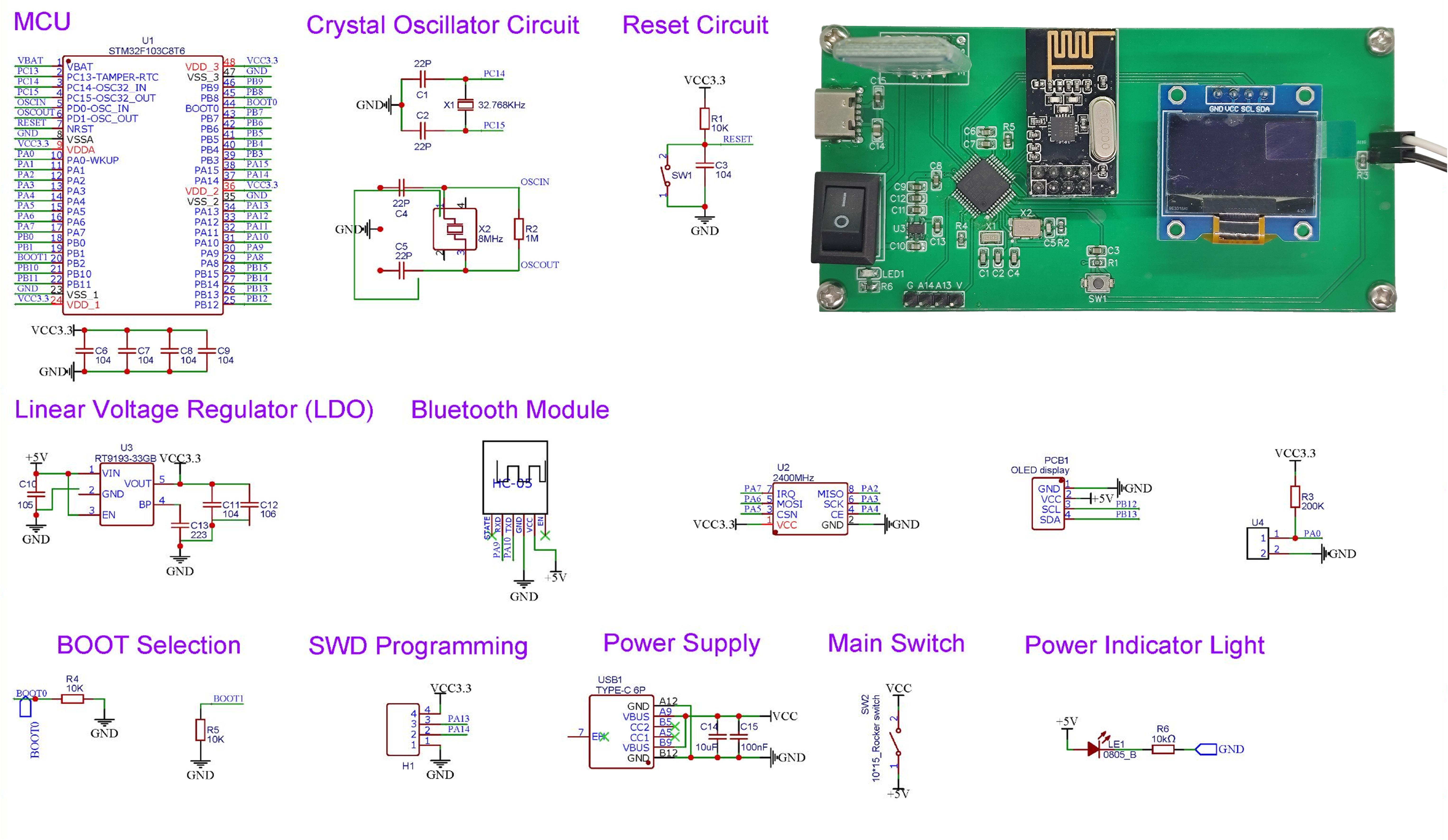


Fig. S33 Circuit board and design of PCB1 used for data processing and signal transmission


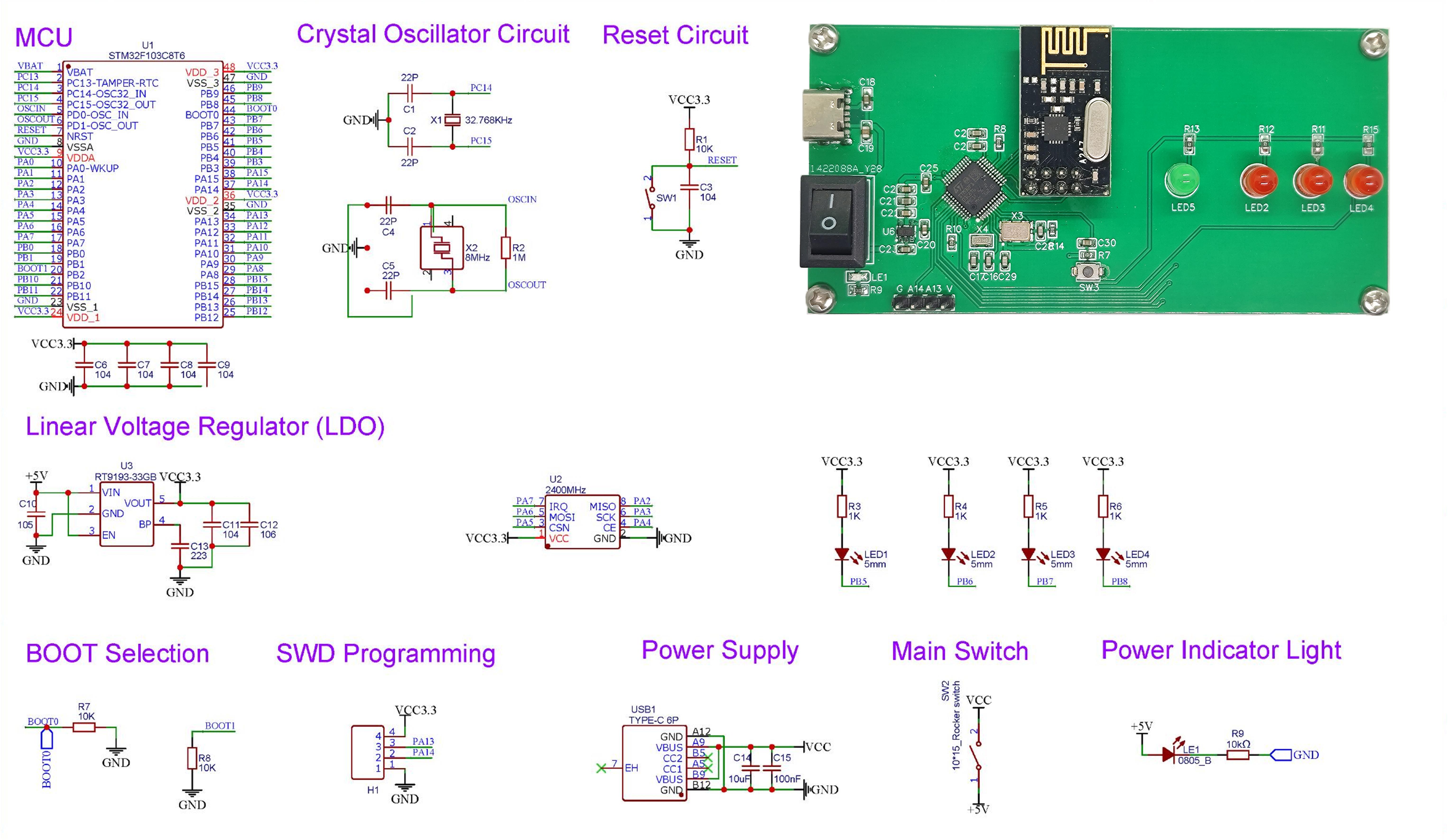


Fig. S34 Circuit board and design of PCB2 used for signal reception


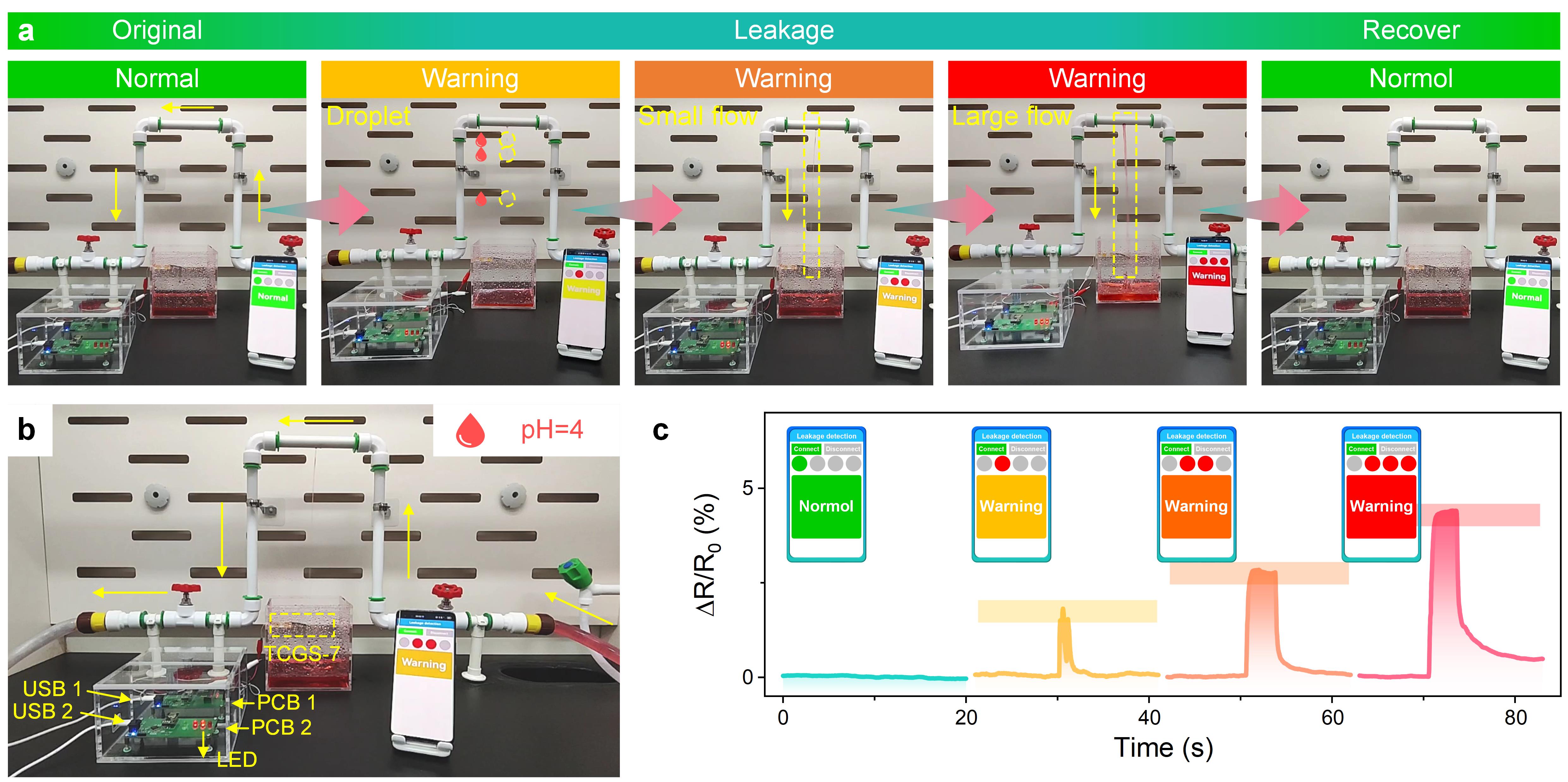


Fig. S35 The leakage detection process for the liquid with pH = 4. **a** Transition from a normal state through droplet leakage, small flow leakage, large flow leakage, and ultimately to recovery. **b** A magnified view of the leakage detection system, including the TCGS-7-based leakage detection device, pipelines, and the liquid with pH = 4 flowing from right to left. **c** Response signals for various states: normal, droplet leakage, small flow leakage, large flow leakage


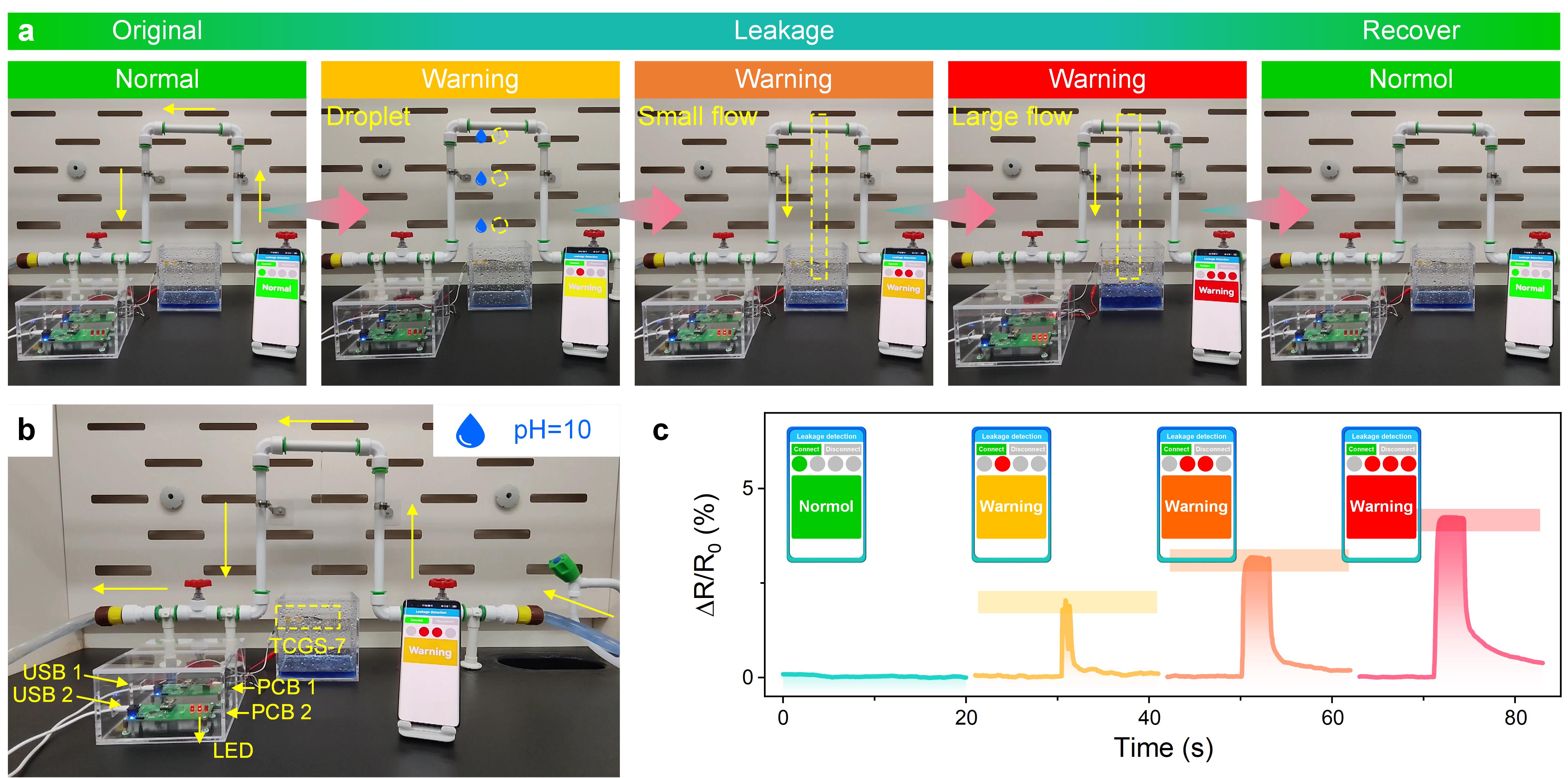


Fig. S36 The leakage detection process for the liquid with pH = 10. **a** Transition from a normal state through droplet leakage, small flow leakage, large flow leakage, and ultimately to recovery. **b** A magnified view of the leakage detection system, including the TCGS-7-based leakage detection device, pipelines, and the liquid with pH = 10 flowing from right to left. **c** Response signals for various states: normal, droplet leakage, small flow leakage, large flow leakage


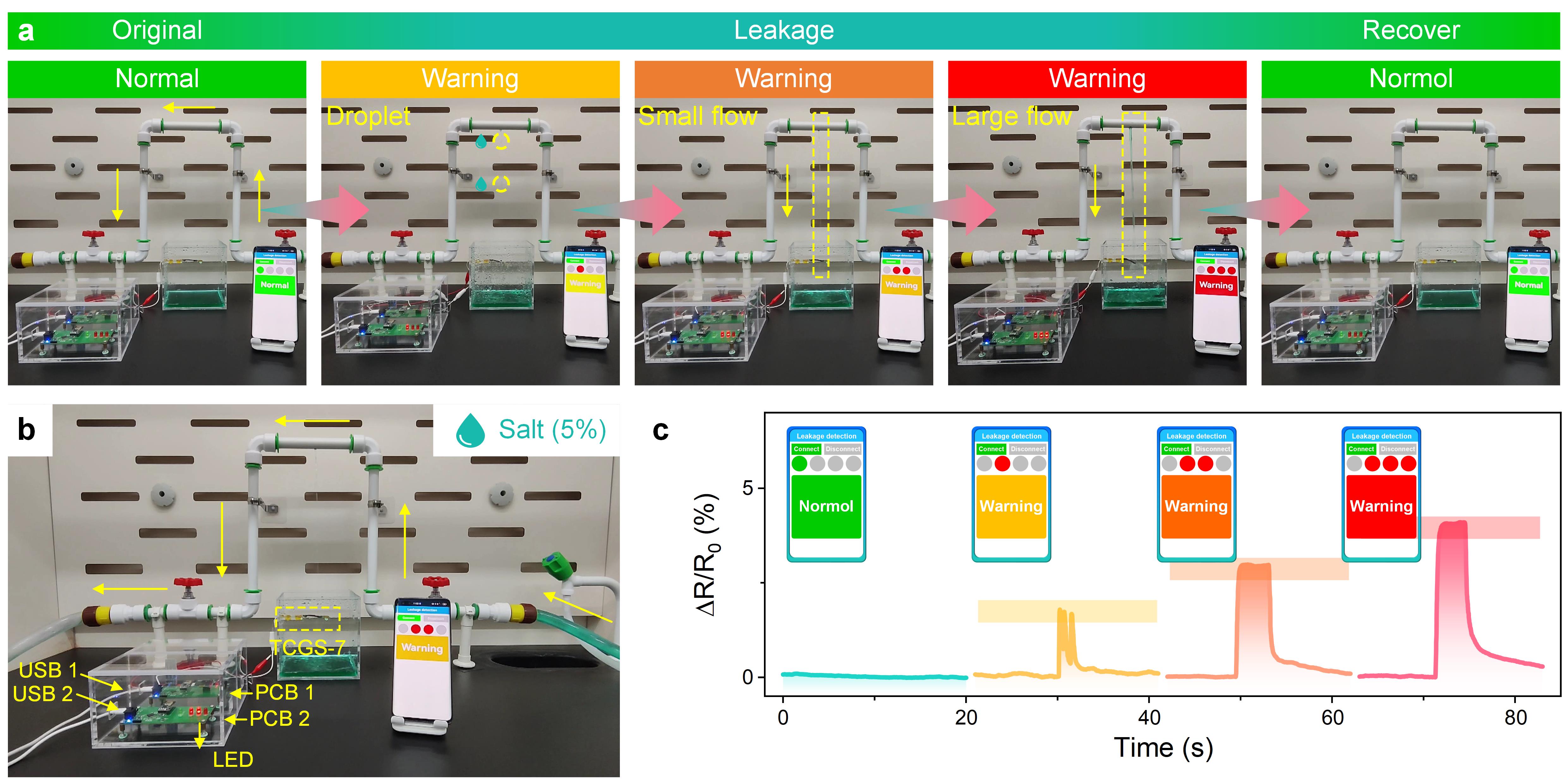


Fig. S37 The leakage detection process for the liquid with 5% salt. **a** Transition from a normal state through droplet leakage, small flow leakage, large flow leakage, and ultimately to recovery. **b** A magnified view of the leakage detection system, including the TCGS-7-based leakage detection device, pipelines, and the liquid with 5% salt flowing from right to left. **c** Response signals for various states: normal, droplet leakage, small flow leakage, large flow leakage


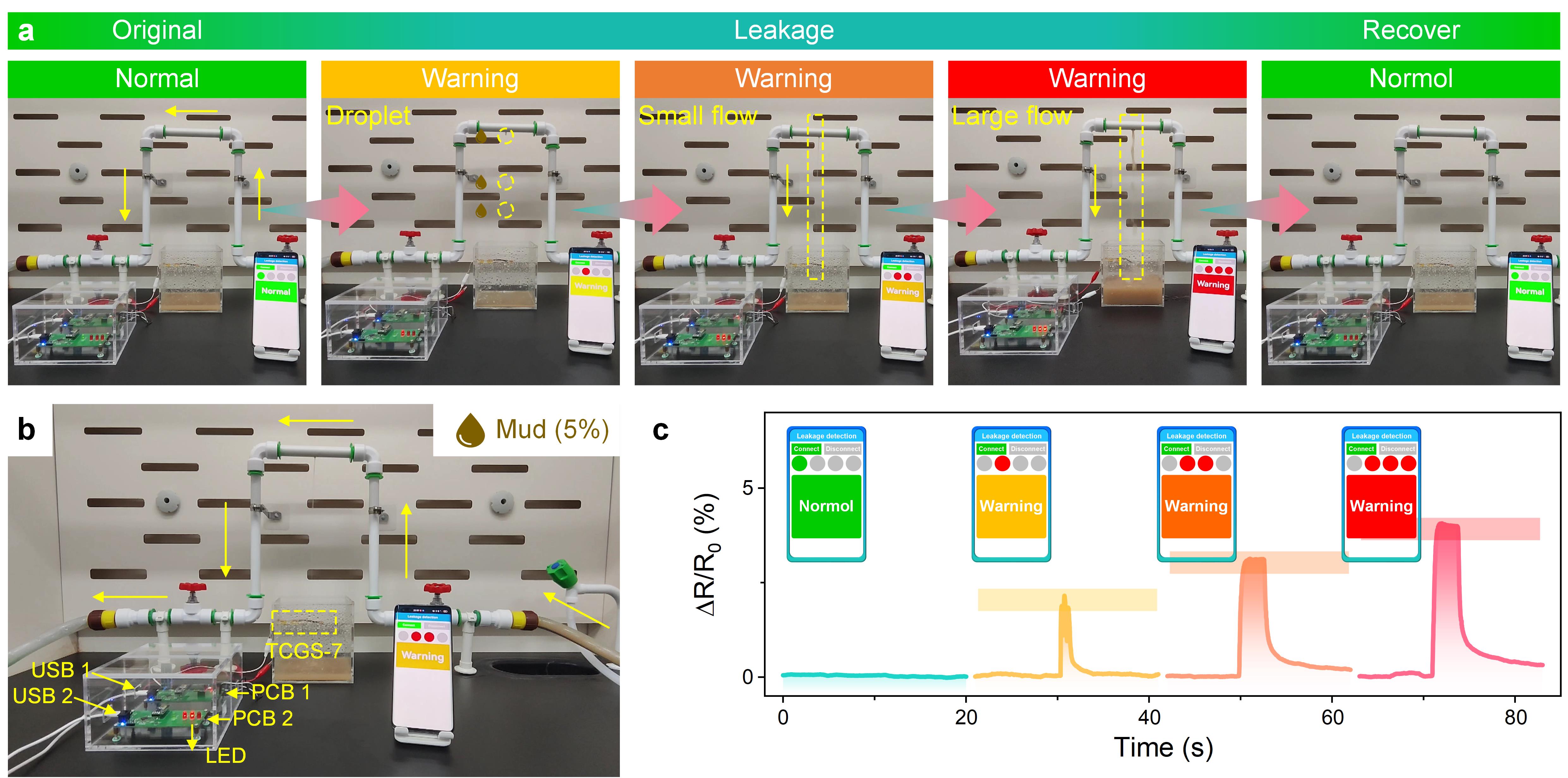


Fig. S38 The leakage detection process for the liquid with 5% mud. **a** Transition from a normal state through droplet leakage, small flow leakage, large flow leakage, and ultimately to recovery. **b** A magnified view of the leakage detection system, including the TCGS-7-based leakage detection device, pipelines, and the liquid with 5% mud flowing from right to left. **c** Response signals for various states: normal, droplet leakage, small flow leakage, large flow leakage

## Supplementary Tables

Table S1 Component content of the TC-$w$

| Sample | TPU (wt%) | Na_2_SO_4_ (wt%) | CNTs (wt%) |  |
| --- | --- | --- | --- | --- |
|  |  |  |  |  |
| TC-0 | 70 | 30 | 0 |  |
| TC-0.5 | 70 | 30 | 0.5 |  |
| TC-1 | 70 | 30 | 1 |  |
| TC-1.5 | 70 | 30 | 1.5 |  |

Table S2 The atomic ratio (C/O) of GNS, TC-1, and TCGS-7

| Sample | C atomic % | O atomic % | C/O atomic ratio |  |
| --- | --- | --- | --- | --- |
|  |  |  |  |  |
| TCGS-7 | 59.2 | 26.41 | 2.24 |  |
| TC-1 | 72.33 | 23.47 | 3.08 |  |
| GNS | 87.62 | 12.38 | 7.08 |  |

**Supplementary Videos**

Video S1 The dynamic process of droplets impacting the TCGS-7 at a velocity of 0.77 m s^–1^

Video S2 The dynamic process of droplets impacting the TCGS-7 at a velocity of 0.99 m s^–1^

Video S3 The dynamic process of droplets impacting the TCGS-7 at a velocity of 1.17 m s^–1^

Video S4 The dynamic process of droplets impacting the TCGS-7 at a velocity of 1.40 m s^–1^

Video S5 The leakage detection process for the liquid with tap water

Video S6 The leakage detection process for the liquid with pH = 4

Video S7 The leakage detection process for the liquid with pH = 10

Video S8 The leakage detection process for the liquid with 5% salt

Video S9 The leakage detection process for the liquid with 5% mud
